# An artificial intelligence model for early-stage breast cancer classification from histopathological biopsy images

**DOI:** 10.3389/frai.2025.1627876

**Published:** 2025-09-12

**Authors:** Neil Chaudhary, A. Z. Dhunny

**Affiliations:** ^1^Pangea Society, New Delhi, India; ^2^Cyber Analytics, Port Louis, Mauritius

**Keywords:** breast cancer classification, histopathological images, deep learning, DenseNet121, multi-scale feature fusion, convolutional neural networks (CNNs), subtype detection, medical image analysis

## Abstract

Accurate identification of breast cancer subtypes is essential for guiding treatment decisions and improving patient outcomes. In current clinical practice, determining histological subtypes often requires additional invasive procedures, delaying treatment initiation. This study proposes a deep learning-based model built on a DenseNet121 backbone with a multi-scale feature fusion strategy, designed to classify breast cancer from histopathological biopsy images. Trained and evaluated on the publicly available BreaKHis dataset using 5-fold cross-validation, the model achieved a binary classification accuracy of 97.1%, and subtype classification accuracies of 93.8% for benign tumors and 92.0% for malignant tumors. These results demonstrate the model’s ability to capture morphological cues at multiple levels of abstraction and highlight its potential as a diagnostic support tool in digital pathology workflows.

## 1 Introduction

Breast cancer remains one of the most prevalent cancers globally, representing a significant health challenge for women across all age groups. According to the World Health Organization (WHO), over 2.3 million women were diagnosed with breast cancer in 2020, making it the most diagnosed cancer worldwide and the leading cause of cancer-related deaths among women (WHO, 2021). The incidence of breast cancer is rising by approximately 3% per year, with higher mortality rates observed in lower-income countries due to limited access to early screening and treatment. In wealthier nations, one in 12 women is diagnosed with breast cancer, whereas in lower-income countries, the rate is one in 27. More concerning is the disparity in mortality: one in 48 women dies from breast cancer in low-income countries compared to one in 71 in high-income countries [World Health Organization (WHO), 2022]. In sub-Saharan Africa, breast cancer now has the highest mortality rate among all cancers affecting women, surpassing cervical cancer. It accounts for 20% of cancer-related deaths in women, with incidence rates varying by region: 30.4 per 100,000 women in Eastern Africa, 26.8 in Central Africa, 38.6 in Western Africa, and 38.9 in Southern Africa. Despite lower incidence rates than in developed countries, the mortality-to-incidence ratio remains alarmingly high at 0.55 in Central Africa, compared to just 0.16 in the United States (GLOBOCAN, 2020).

Early detection has been identified as a critical factor in improving survival rates, with studies showing that early-stage breast cancer has a 90% 5-year survival rate compared to late-stage diagnoses, which can drop to 27% (Siegel et al., 2022). This significant effort is to enhance diagnostic techniques to detect breast cancer earlier and more accurately. Over the past decade, there have been remarkable advances in breast cancer detection, particularly with the integration of advanced imaging techniques and machine learning. Traditional imaging modalities, such as mammography and ultrasound, have been instrumental in identifying breast lesions; however, their sensitivity can decrease in women with dense breast tissue (Kolb et al., 2002). Magnetic resonance imaging (MRI) has emerged as a superior modality for detecting early-stage breast cancer due to its high spatial resolution and ability to differentiate between benign and malignant lesions (Kuhl et al., 2005). Despite its advantages, retention remains complex and prone to variability among radiologists, emphasizing the need for more standardized and accurate approaches. Artificial intelligence (AI) models present a transformative solution to these challenges, especially in resource-limited settings. By leveraging AI to analyze biopsy images, diagnostic accuracy can be improved while reducing the workload of overburdened pathologists. AI-powered systems have demonstrated sensitivities of up to 94.5% in detecting malignant tumors in histopathological images, reducing false-negative rates significantly (Esteva et al., 2021). Additionally, AI-assisted diagnostics have the potential to cut down diagnosis time from weeks to just hours, allowing for earlier treatment initiation and improved patient outcomes. This is particularly beneficial in low-resource settings where a shortage of trained pathologists delays cancer detection (McKinney et al., 2020). By integrating AI into breast cancer diagnostics, healthcare systems in developing countries can bridge the gap in early detection, reduce misdiagnosis rates, and ultimately lower breast cancer mortality. Scalable AI solutions, combined with improved screening programs and public awareness efforts, have the potential to significantly enhance cancer care worldwide.

Despite significant progress in imaging modalities and AI-driven diagnostic tools, most existing models focus primarily on binary classification—differentiating benign from malignant tumors—without addressing the more nuanced and clinically challenging task of histological subtype classification. This limitation hinders their real-world applicability, particularly in cases where treatment decisions depend on precise subtype identification. These gaps underscore the need for a more robust, subtype-aware AI framework trained on whole-slide biopsy images to improve diagnostic granularity and clinical utility.

### 1.1 Brief review of artificial intelligence in breast cancer

This section provides a brief literature review of what technology has been used for the detection of breast cancer over the past few years for breast cancer detection. And this is going to justify our innovative model and method in the further sections.

AI has revolutionized the healthcare landscape, offering transformative capabilities in automating and enhancing diagnostic processes. In the context of breast cancer detection, AI models have demonstrated superior performance in analyzing complex MRI datasets, identifying patterns that may elude human experts (Lehman et al., 2019). For instance, deep learning convolutional neural networks (CNNs) and attention mechanisms have been applied to segment breast tissues, classify lesions, and predict malignancy with high accuracy (Litjens et al., 2017). These advancements not only reduce errors but also minimize the workload of radiologists, making AI a valuable tool in clinical settings. The integration of AI into breast cancer detection workflows has also been shown to address the challenges of class imbalance and variability in imaging quality. Many studies have employed transfer learning and ensemble techniques to overcome these challenges, achieving higher sensitivity and specificity than traditional methods (McKinney et al., 2020). Moreover, AI-driven algorithms can process datasets quickly and consistently, offering significant advantages in population-based breast cancer screening programs (Rodrigues et al., 2021).

Kaymak et al. (2017) applied neural networks to classify 176 histopathology images, using discrete Haar wavelets for preprocessing. Radial Basis Function Networks outperformed Back Propagation Networks, achieving 70.49% accuracy. Fu et al. (2022) predicted Peripherally Inserted Central Catheter (PICC)-related thrombosis in breast cancer patients using Artificial Neural Network (ANN) and Synthetic Minority Over-sampling Technique (SMOTE) to address class imbalance, outperforming logistic regression (AUC: 0.742 vs. 0.675). Punitha et al. (2021) combined Artificial Immune Systems and Bee Colony optimization for feature selection, improving multilayer perceptron (MLP) performance on the Wisconsin Breast Cancer Dataset (WBCD) dataset for automated diagnosis.

Sharma et al. (2024) used the WBCD to build a stacked ensemble framework integrating Decision Tree, AdaBoost, Gaussian Naive Bayes, and MLP classifiers, achieving 97.66% accuracy. Ensemble learning and feature engineering contributed to its robust performance. Liu et al. (2024a) enhanced a Visual Geometry Group 16-layer network (VGG16)-based model with transfer learning and focal loss to handle class imbalance in 7,841 mammograms, yielding a 96.95% accuracy. Li et al. (2024b) proposed a multimodal fusion model that combined ResNet34-extracted MRI features with RNA-seq gene expression data, using transformers and attention mechanisms to predict treatment response in breast cancer patients more accurately than single-modality approaches.

Munshi et al. (2024) introduced a novel breast cancer detection method by combining an optimized ensemble learning framework with explainable AI (XAI). Using the Wisconsin Breast Cancer Dataset, which contains 32 numerical features, they employed an ensemble model integrating CNNs with traditional machine learning algorithms like random forest (RF) and support vector machine (SVM). U-NET was used for image-based tasks, and a voting mechanism combined RF and SVM predictions. The model achieved an impressive accuracy of 99.99%, surpassing state-of-the-art methods in precision, recall, and F1-score. The integration of XAI enhanced the interpretability and transparency of model decisions, further improving breast cancer diagnostics.

Atrey et al. (2024) developed a multimodal classification system for breast cancer by integrating mammogram (MG) and ultrasound (US) images using deep learning and traditional machine learning techniques. The dataset, sourced from AIIMS, Raipur, India, consisted of 31 patients and 43 MG and 43 US images, which were augmented to 1,032 images. Data preprocessing involved manual region-of-interest (ROI) annotation by radiologists and noise filtering techniques for both image types. The deep learning model, ResNet-18, was used for feature extraction, while SVM classified the fused features. The hybrid ResNet-SVM approach achieved a classification accuracy of 99.22%, significantly outperforming unimodal methods. This study demonstrates the effectiveness of combining deep learning with traditional machine learning for improved breast cancer diagnosis.

Robinson and Preethi (2024) developed the SDM-WHO-RNN model, combining fully convolutional networks with RNNs for classifying 9,109 histopathological images from the Breast Cancer Histopathological Image (BreaKHis) and BH datasets. Preprocessing steps included resizing, noise reduction, and stain normalization, while Least Squares Convolutional Encoder-Decoder (LS-CED) was used for tumor segmentation. The system achieved 97.9% accuracy in detecting complex tumor cells. Zhang et al. (2024) proposed the Convergent Difference Neural Network (V-CDNN), an ensemble of Convergent Difference Neural Networks optimized with a Neural Dynamic Algorithm and softsign activations. Trained on public datasets, the model reached a perfect 100% classification accuracy, demonstrating both speed and robustness.

In their 2021 study, Meha Desai and Manan Shah compared the effectiveness of MLP and CNN for breast cancer detection. The study used datasets such as BreakHis, WBCD, and WDBC, with images and biomarkers preprocessed through normalization and feature extraction (e.g., Discrete Cosine Transform). While both MLP and CNN were trained to classify breast abnormalities, CNN consistently outperformed MLP in terms of accuracy. CNN achieved ~99.86% accuracy on the BreakHis dataset, while MLP’s performance was generally lower. CNN was found to be more reliable for image-based diagnosis, whereas MLP showed limitations for larger datasets.

Further to this, Ezzat et al. (2023) proposed an optimized Bayesian convolutional neural network (OBCNN) for detecting invasive ductal carcinoma (IDC) from histopathology images. This approach integrated ResNet101V2 with Mucinous Carcinoma (MC) for uncertainty estimation. Using the slime mould algorithm to optimize dropout rates, the study fine-tuned the architecture, outperforming other pre-trained networks such as VGG16 and DenseNet121. The model demonstrated significant robustness in diagnosis and generalization. Joseph et al. (2022) proposed a multi-classification approach for breast cancer using handcrafted features, including Hu moments, Haralick textures, and color histograms. These features were combined with deep neural networks trained on the BreakHis dataset. Data augmentation was applied to address overfitting, and a four-layer dense network with softmax activation was employed to enhance classification accuracy.

Paul et al. (2023) proposed a novel breast cancer detection system using an SDM-WHO-RNN classifier combined with LS-CED segmentation. Their approach incorporated preprocessing steps, such as noise elimination and image normalization. The LS-CED segmentation localized nuclei, which were then classified based on size and shape. The system demonstrated high accuracy in detecting cancerous cells. Taheri and Omranpour (2023) introduced an ensemble meta-feature generator (EMFSG-Net) for classifying ultrasound images. Leveraging transfer learning with the VGG-16 architecture, the model employed support vector regression (SVR) to create efficient feature spaces. To address issues like overfitting and dead neurons, the leaky-ReLU activation function was incorporated, leading to improved feature representation and classification performance. Muduli et al. (2021) proposed a deep CNN for automated breast cancer classification using mammograms and ultrasound images. The model, consisting of five learnable layers, employed manual cropping for feature extraction and data augmentation to improve generalization. The approach achieved superior results compared to state-of-the-art methods.

Dr. Suvidha Tripathi’s research contributes significantly to the field of breast cancer histopathology image classification, particularly through innovative approaches that integrate spatial context and hybrid feature representations. In one study, Tripathi et al. proposed a BiLSTM-based patch modeling framework, which treats histopathology image patches as sequential data to capture spatial continuity—an aspect often overlooked in conventional CNNs. This model demonstrated strong performance on the Breast Cancer Histology (Grand Challenge 2018 dataset), highlighting the value of contextual learning in improving classification accuracy. In subsequent work, she introduced a hybrid architecture combining CNNs with Bag-of-Visual-Words (BoVW), effectively integrating handcrafted and deep features to enhance discrimination in limited data settings. This approach outperformed standard deep networks like ResNet and DenseNet on the same dataset, underscoring the benefits of feature selection and hybrid modeling in medical imaging tasks. Together, these studies emphasize alternative paths for improving classification performance—either by modeling inter-patch relationships or by enriching feature spaces—both of which are highly relevant for advancing automated diagnosis in breast cancer histopathology.

This research is toward an innovative approach for breast cancer detection, including the multiple subclasses of breast cancer, which still confuses medical doctors, and it hence makes patients undergo more advanced invasive tests for a better understanding of the disease. The technique is based on an AI model trained on a sizable batch of data. Section 2 discusses the model in detail, Section 3 shows the Results and Discussion, and the Conclusion is given in Section 4.

## 2 Methodology

This methodology section combines an explanation of the different types of breast cancer and their subclasses, along with an advanced AI technique which has been developed to analyse breast cancer results of biopsy. The aim is, instead of making women go again and again through trial and error with treatments or misdiagnosing the cancer, using AI can make all the difference. As mentioned in the literature review section above, AI tech has changed the way the world views medicine now. Therefore, in this study, we have designed a powerful tool for diagnosing the multiple subclasses. This goes toward a humanitarian cause as women are the target here.

Benign and malignant breast cancers differ in their behavior, prognosis, and treatment approach. Benign tumors, such as fibroadenomas or cysts, are non-cancerous growths that do not invade surrounding tissues or spread to other parts of the body. They tend to have well-defined borders, grow slowly, and usually pose little to no health risk. In contrast, malignant tumors are cancerous and have the potential to invade nearby tissues and metastasize to distant organs through the lymphatic system or bloodstream (Robbins et al., 2010). Malignant breast cancers, such as IDCs, invasive lobular carcinoma (LCs), etc., show uncontrolled cell growth and can require serious medical intervention in the form of surgery and/or radiation therapy. Early identification of a tumour as benign or malignant is critical to deliver appropriate treatment and improve patient health (National Cancer Institute, 2021). In histopathological images, benign and malignant tissues exhibit discernibly unique optical features. Benign tumours present as well-organized structures consisting of cells with uniform shapes, minimal mitotic activity, and intact basement membranes. The stromal and glandular components of benign lesions are usually preserved, showing regular nuclei and minimal pleomorphism. On the other hand, malignant lesions show irregular cellular arrangements, pleomorphic nuclei and frequent mitotic figures (Robbins et al., 2010). They often display disrupted basement membranes and an increased frequency of necrotic regions, further highlighting their aggressive nature. AI image analysis of biopsy images will be able to use these intrinsic differences to ensure earlier and more reliable classification into benign and malignant cases (Rakhlin et al., 2018).

**Figure 1:**
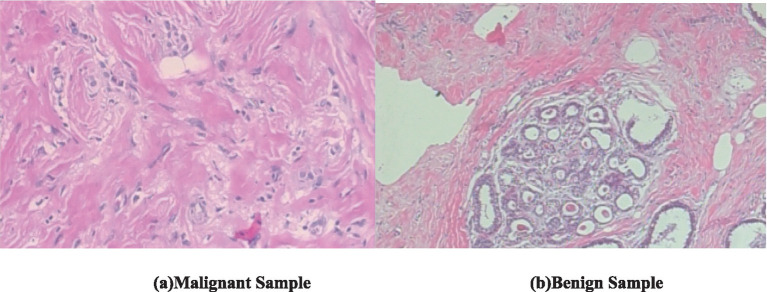
Biopsy image of malignant and benign sample (BreakHis dataset). **(a)** Malignant sample. **(b)** Benign sample.

The visual features in the biopsy images provide key characteristics for distinguishing malignant and benign breast tissues. Figure 1a (malignant sample) exhibits irregular, disorganized cell structures with pleomorphic nuclei and a dense, fibrous stroma, indicating uncontrolled cancerous growth. The nuclei appear darker and more varied in shape, with increased mitotic activity and loss of structural integrity (Robbins et al., 2010). In contrast, Figure 1b (benign sample) shows well-organized, rounded glandular structures surrounded by normal fibrous tissue, with uniform cell shapes and minimal pleomorphism (National Cancer Institute, 2021). Both these images have a magnification of 100x.

### 2.1 Dataset

In this study, we utilize the BreaKHis dataset compiled by Spanhol et al. (2016), which contains 9,109 microscopic images of breast tissue captured at four magnification levels: 40×, 100×, 200×, and 400×. These images are categorized into two overarching classes—benign and malignant tumors. Within each class, the dataset further identifies four subtypes: for benign tumors, these include adenosis (A), fibroadenoma (F), phyllodes tumor (PT), and tubular adenoma (TA); and for malignant tumors, ductal carcinoma (DC), LC, mucinous carcinoma (MC), and papillary carcinoma (PC). The image filenames in the BreaKHis dataset encode multiple details about each sample, including the biopsy method used, the tumor’s classification (benign or malignant), its histological subtype, the patient ID, and the magnification level. For instance, the file named SOB_B_TA-14-4659-40-001.png refers to the first image of a benign tumor classified as TA, obtained from patient 14-4659 at a magnification of 40× using the Segmental Orthogonal Biopsy (SOB) technique (Spanhol et al., 2016).

It comprises samples from 82 patients collected at a single institution in Brazil, with no publicly available metadata on age, ethnicity, or receptor status—24 with benign tumors and 58 with malignant tumors. Of the 7,909 images used in the final analysis, 5,429 are malignant and 2,480 are benign, distributed across the aforementioned magnification levels. Following preprocessing and class balancing, the revised class distribution is outlined in Table 1.

**Table 1:** Dataset image count for each cancer type.

Cancer type	Final image count	Subtype
Adenosis	444	Benign
Fibroadenoma	730	Benign
Phyllodes tumor	453	Benign
Tubular adenoma	569	Benign
Ductal carcinoma	797	Malignant
Lobular carcinoma	626	Malignant
Mucinous carcinoma	792	Malignant
Papillary carcinoma	560	Malignant

Benign tumors, by histological standards, lack features associated with malignancy, such as cellular atypia, mitotic activity, invasive growth, or metastatic potential. They typically exhibit localized, slow growth and are considered non-lethal (Robbins and Cotran, 2010). In contrast, malignant tumors are characterized by their capacity to invade neighboring tissues and metastasize to distant sites, often posing life-threatening risks (Robbins and Cotran, 2010). All tissue samples in this dataset were acquired through SOB—a surgical procedure also referred to as partial mastectomy or excisional biopsy. Unlike needle-based techniques, SOB yields larger tissue specimens and is generally performed under general anesthesia in a clinical setting (American Cancer Society, 2019). The classification of tumors into distinct subtypes is based on their cellular morphology under microscopic examination, which holds significance for both prognosis and treatment planning (National Cancer Institute, 2021).

The BreakHis dataset was used for both binary (benign vs. malignant) and multiclass subclass classification. All histopathological images were resized to 128 × 128 pixels and normalized to a standard RGB range. A stratified 80:20 train-test split was employed to preserve class balance across sets. For cross-validation, a 5-fold split was used with data shuffling to ensure unbiased performance evaluation, and no image was shared across folds.

Prior to splitting, real-time augmentation was applied separately to the benign and malignant image sets, increasing the number of benign samples to 7,440 and malignant samples to 9,385. The augmented dataset, totaling 16,825 images, was then split into training, validation, and test sets using a stratified class-wise approach. For both benign and malignant classes, 20% of the data was reserved for testing, and the remaining 80% was further divided in a 75:25 ratio to form the training and validation sets. This resulted in 10,095 images in the training set and 3,365 images each in the validation and test sets.

To improve generalization and address the risk of overfitting due to limited data, we implemented an extensive on-the-fly augmentation pipeline using TensorFlow’s data generators. This included spatial and color transformations to simulate natural histological variation while preserving key tissue features. Table 2 summarizes the augmentation parameters used during training.

**Table 2:** Augmentation parameters.

Augmentation type	Range/value
Rotation	90°, 180°, 270°
Horizontal flip	Random
Vertical flip	Random
Zoom	Up to ±10%
Shear	±10°
Brightness adjustment	±20% range
Contrast adjustment	±20% range
Saturation/hue jitter	Applied with random factor

These augmentations were applied to each batch during training to expose the model to diverse variations without expanding the dataset size. In addition, we used early stopping (patience = 5) and ReduceLROnPlateau scheduling to prevent overfitting, along with dropout (0.45), L2 kernel regularization, and batch normalization in each dense layer. Learning curves were generated during each fold to monitor training behavior. The curves showed a minimal gap between training and validation accuracy, indicating stable convergence and effective generalization across classes.

To address class imbalance—particularly for underrepresented subtypes, such as PT and LC—we applied targeted data augmentation during preprocessing. For most classes, each image was augmented using 90° and 180° rotations along with horizontal flipping, effectively tripling their sample size. However, for class label 4 (DC), which was already sufficiently represented, only minimal augmentation was applied to prevent overrepresentation. This strategy helped balance the dataset and reduce bias during training, without altering the loss function or applying weighted sampling.

### 2.2 Artificial intelligence model

**Figure 2:**
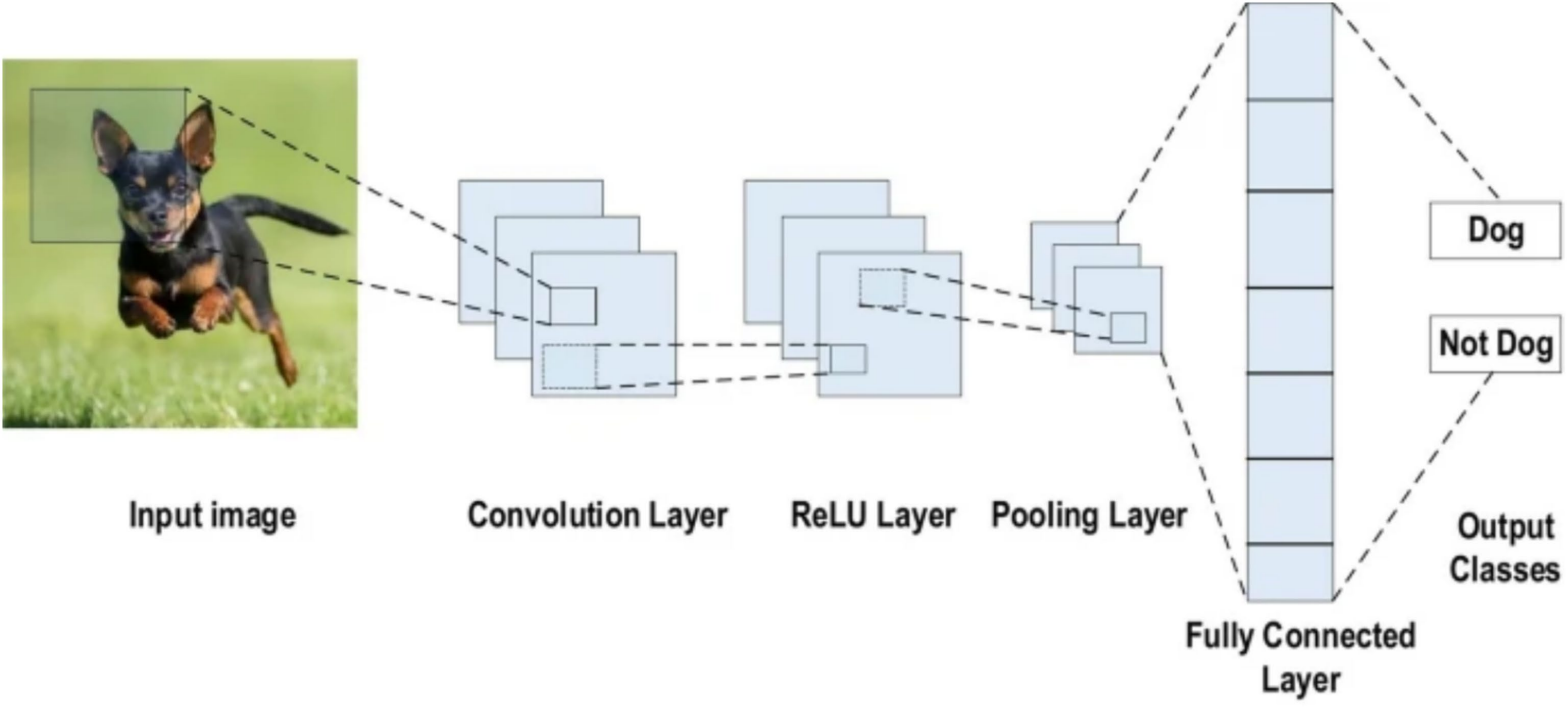
CNN architecture (Alzubaidi et al., 2021).

A CNN is a deep learning architecture tailored for grid-like data such as images, leveraging spatial hierarchies to extract local and global features (LeCun et al., 1998). Input images are processed through convolutional layers that apply learnable filters to detect features like edges and textures, generating spatially preserved feature maps (Krizhevsky et al., 2012). Non-linear activation functions, typically ReLU (Glorot et al., 2011), introduce complexity, while pooling layers reduce spatial dimensions and enhance translational invariance (Scherer et al., 2010). Deeper layers capture higher-level abstractions, which are then passed to fully connected layers for classification (Simonyan and Zisserman, 2014). Enhancements such as batch normalization, residual connections (He et al., 2016), and attention mechanisms have further advanced CNN performance, particularly in medical image analysis (see Figure 2).

#### 2.2.1 Classifying images as benign or malignant

In the first approach, a conventional CNN architecture was developed to perform a binary classification of histopathological breast cancer images into benign and malignant categories. Rather than selecting parameters arbitrarily, several architectural configurations were tested iteratively to arrive at a structure that balanced computational efficiency and classification performance. The final model comprised three convolutional layers with 16, 32, and 16 filters, respectively, employing 4 × 4 kernels to effectively capture both low- and mid-level spatial features. This configuration aligns with literature that emphasizes the benefit of using multiple convolutional layers of varying depth for feature extraction in histological images (Spanhol et al., 2016; Bayramoglu et al., 2017). ReLU activation was applied after each convolutional layer to introduce non-linearity, mitigate vanishing gradients, and support efficient learning. MaxPooling layers followed each convolutional block to reduce spatial dimensions and retain salient features (Rakhlin et al., 2018).

**Figure 3:**
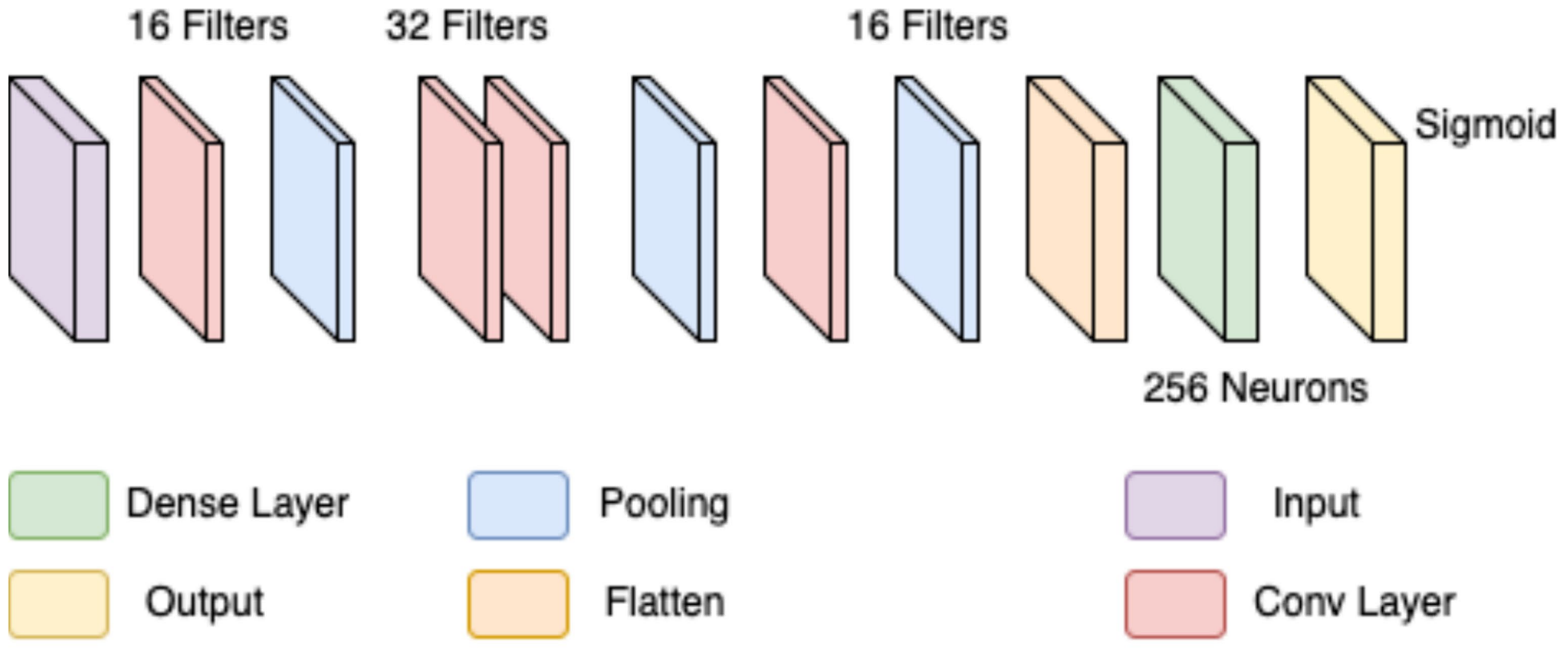
Initial CNN.

The output of the convolutional stack was flattened and passed into a fully connected layer with 256 neurons, enabling high-capacity representation before final classification. A sigmoid-activated output layer was used for binary probability prediction, and binary cross-entropy served as the loss function, both standard choices in binary medical image classification tasks (Gandomkar et al., 2018; Liu et al., 2019). The model was trained using the Adam optimizer (initial learning rate = 0.0001) over 20 epochs, leveraging its adaptive learning rate to ensure stable convergence in a data-limited environment (Bandi et al., 2018). Despite sound architecture and training configuration, this initial model was limited by two key issues: a substantial class imbalance skewed toward malignant samples, and the failure to account for magnification-level heterogeneity across the dataset (40×, 100×, 200×, and 400×). This led to model overfitting, poor generalization, and relatively low accuracy and F1-scores on the validation set. The architectural layout is depicted in Figure 3 (see Table 3).

**Table 3:** Hyperparameters for initial binary classification CNN.

Hyperparameter	Value	Explanation
Convolutional filters	16, 32, 16	The number of filters in each convolutional layer. More filters allow for learning more complex features but increase computational cost.
Kernel size	4×4	Defines the size of the filter that scans the image. A smaller kernel captures fine details, while a larger one captures broader patterns.
Activation function	ReLU	Introduces non-linearity to the network, allowing it to learn complex patterns. ReLU prevents the vanishing gradient problem.
Pooling type	MaxPooling	Reduces spatial dimensions, retaining the most important features while lowering computational cost and reducing overfitting.
Fully connected layer	256 neurons	Processes extracted features and maps them to the final classification decision. A higher number of neurons allows for better learning capacity.
Output activation	Sigmoid	Outputs a probability score between 0 and 1, making it suitable for binary classification tasks.
Loss function	Binary cross-entropy	Measures how well the predicted probabilities match the true labels, ensuring optimal training for binary classification.
Optimizer	Adam	Adjusts learning rates dynamically for each parameter, leading to faster convergence and better training stability.
Epochs	20	The number of times the model passes through the entire dataset during training. More epochs improve learning but may cause overfitting.

**Figure 4:**
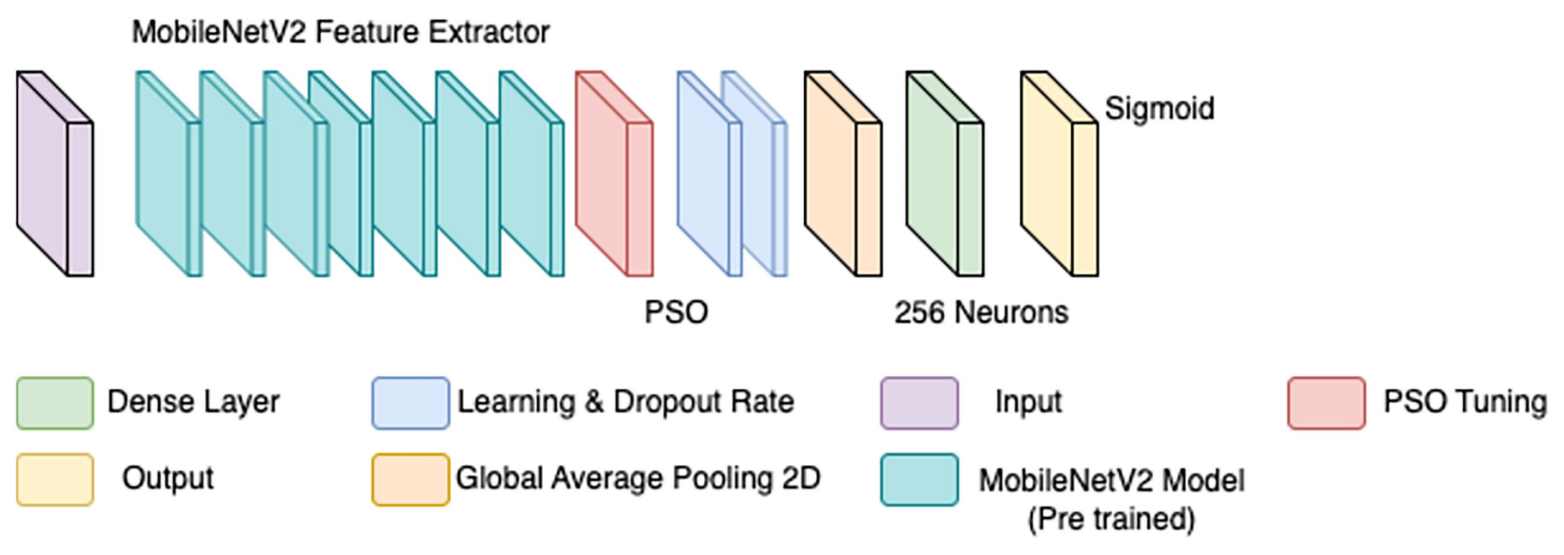
Updated CNN.

To overcome the limitations of the initial CNN model, a refined approach was adopted, incorporating particle swarm optimization (PSO) for hyperparameter tuning. PSO has demonstrated effectiveness in medical imaging by dynamically optimizing parameters for improved convergence (Houssein et al., 2021). Rather than training a model from scratch, MobileNetV2—a lightweight, pre-trained CNN trained on ImageNet—was employed as a base feature extractor (illustrated by the teal blocks in Figure 4). Transfer learning with pre-trained models has been shown to enhance performance in histopathological classification tasks, especially when annotated datasets are limited (Srinivasu et al., 2021).

The BreaKHis dataset was stratified by magnification level (40×, 100×, 200×, and 400×), and separate models were trained for each resolution, aligning with evidence that such granularity improves classification performance in multi-resolution tasks (Bayramoglu et al., 2017). Within each subset, class balance was ensured by maintaining 250–350 images per class. Data augmentation was applied using ImageDataGenerator, with transformations including rescaling (1./255), width and height shifts (0.2), shear (0.2), zoom (0.2), and horizontal flips. These augmentations were crucial for improving generalization in data-scarce conditions (Bandi et al., 2018).

PSO was used to fine-tune key hyperparameters—specifically, learning rate (searched in the range 1e-5 to 1e-2) and dropout rate (0.3–0.7)—to enhance convergence and prevent overfitting. Optimization was first performed on the 40 × magnification model; the resulting best parameters were then transferred to models for the other magnifications. To further stabilize training, a ReduceLROnPlateau scheduler (factor = 0.5, patience = 3, min_lr = 1e-6) dynamically adjusted the learning rate based on validation loss trends, while EarlyStopping (patience = 5, restore_best_weights = true) halted training when no improvement was observed, thereby avoiding overfitting and saving compute resources (Srinivasu et al., 2021). The final architecture is illustrated in Figure 4. Hyperparameters for this architecture are in Table 4.

**Table 4:** Hyperparameters for the first attempt of per-magnification training.

Hyperparameter	Value(s) used	Explanation
Optimizer	Particle swarm optimization (PSO)	Instead of using Adam, PSO dynamically selects the best learning rate and dropout rate by minimizing validation loss. This helps in fine-tuning model performance more efficiently.
Learning rate	Optimized by PSO (range: 1e-5 to 1e-2)	Controls the step size of weight updates. A smaller learning rate ensures stable learning, while a larger one speeds up convergence but risks overshooting the optimal point.
Dropout rate	Optimized by PSO (range: 0.3–0.7)	Dropout prevents overfitting by randomly deactivating neurons during training. The PSO algorithm selects the best dropout rate for optimal generalization.
Batch size	16	The number of images processed at once during training. A smaller batch size helps conserve memory but may result in higher training variance.
Input image size	(128, 128, 3)	The input images are resized to 128 × 128 pixels with three color channels to ensure uniformity and reduce computational complexity.
Base model	MobileNetV2 (pre-trained on ImageNet)	A lightweight deep learning model optimized for mobile and embedded vision applications. The feature extraction layers are frozen to retain learned representations.
Magnification-based training	40×, 100×, 200×, and 400 × (trained separately)	Instead of training all images together, the dataset is split based on magnification levels to improve feature learning at each resolution.
Data augmentation	Rescaling (1./255), width and height shifts (0.2), shear (0.2), zoom (0.2), and horizontal flips	Artificially increases dataset size by applying transformations, helping the model generalize better to unseen samples.
Validation split	20%	A portion of the dataset is reserved for validation to evaluate model performance and prevent overfitting.
Loss function	Binary cross-entropy	Since the classification is binary (benign vs. malignant), binary cross-entropy is used to compute the error between predicted and actual labels.
Activation function	ReLU (hidden layers), sigmoid (output layer)	ReLU helps prevent vanishing gradients in hidden layers, while Sigmoid is used in the output layer for binary classification (probabilities between 0 and 1).
ReduceLROnPlateau	Factor = 0.5, patience = 3, min_lr = 1e-6	Reduces the learning rate if validation loss stops improving for three consecutive epochs, preventing unnecessary weight updates.
EarlyStopping	Monitor = val_loss, patience = 5, restore_best_weights = true	Stops training if validation loss does not improve for five consecutive epochs, preventing overfitting and saving computation time.
Epochs	30	The number of times the model sees the entire dataset during training. Early stopping ensures the model does not train longer than necessary.

However, this approach too yielded an accuracy far below acceptable levels. F1-scores, recall and precision also hovered around the 50% mark, forcing another change in approach.

The classification of breast cancer histopathology images involved separating the dataset based on different magnification levels (40×, 100×, 200×, and 400×). While this method aimed to leverage magnification-specific features, it resulted in severe dataset fragmentation. Each individual model was trained on a significantly smaller subset of images, which limited the ability to generalize across different test samples. Even with extensive data augmentation, the number of available images remained insufficient, leading to high variance and suboptimal performance during evaluation. Additionally, the previous models relied on relatively shallow CNNs for feature extraction. These networks struggled to effectively capture both low-level and high-level representations within the histopathology images, leading to limited discriminatory power when distinguishing between benign and malignant samples. Given these challenges, a more robust and generalized model was required to improve classification performance across all magnification levels. To overcome the limitations of dataset fragmentation and poor feature extraction, a new model was developed using DenseNet121 as the backbone for feature extraction. DenseNet121, a deep CNN pre-trained on the ImageNet dataset, was chosen due to its efficient parameter utilization and ability to capture intricate hierarchical features. Unlike the previous approach, this model does not separate images based on magnification, allowing for a larger and more diverse training dataset, thereby improving generalization. The proposed model extracts features from three different depths of DenseNet121: conv3_block12_concat, conv4_block24_concat, and conv5_block16_concat. These layers correspond to different levels of abstraction within the network, ensuring that both fine-grained and high-level morphological characteristics are captured. The selection of these layers allows for multi-scale feature extraction, improving the model’s ability to differentiate between benign and malignant samples. The extracted features are processed through a structured pipeline to refine and enhance their representation before classification.

Feature extraction in this model occurred at three levels. The lowest-level feature map, obtained from conv3_block12_concat, captures fundamental visual characteristics such as edges, textures, and color distributions. These features are crucial for identifying initial structural differences in histopathology images, such as variations in cellular arrangements. The mid-level feature map, extracted from conv4_block24_concat, represents more complex patterns, including structural organization within the tissue and variations in gland formation. These features help differentiate between normal and abnormal cellular arrangements. The highest-level feature map, obtained from conv5_block16_concat, provides a more abstract representation, focusing on morphological changes indicative of malignancy, such as nuclear pleomorphism, mitotic activity, and stromal alterations. By incorporating multiple feature extraction levels, the model ensures a comprehensive understanding of tissue characteristics rather than relying on a single-layer representation. To ensure a balance between computational efficiency and adequate feature resolution, an input image size of 128 × 128 was chosen over 256 × 256. While higher resolutions like 256 × 256 could preserve more fine-grained tissue structures, they also significantly increase memory usage and computational load. Given that histopathology images already exhibit high variability and detailed cellular patterns, 128 × 128 provides a sufficient level of detail for feature extraction while allowing for larger batch sizes, faster training, and reduced GPU memory constraints. This choice is particularly important when training multiple models, as excessive computational demands could slow down hyperparameter tuning and optimization.

**Figure 5:**
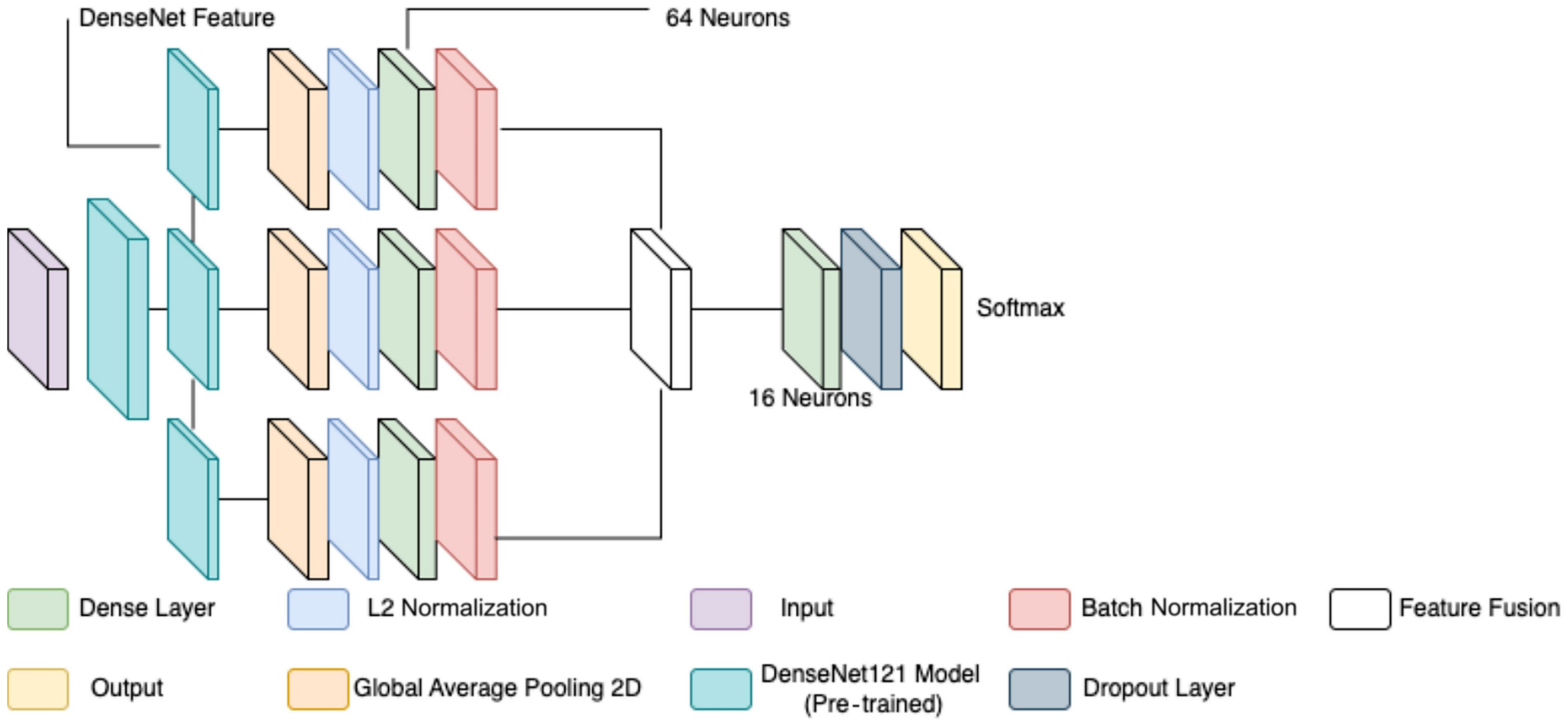
Final binary class CNN.

Each extracted feature map undergoes global average pooling (GAP) to reduce dimensionality while retaining essential spatial information. Unlike max pooling, which selects only the most prominent activations, GAP ensures that all features contribute proportionally to the final representation, enhancing robustness and stability. After pooling, L2 normalization is applied to ensure numerical stability and prevent dominance of certain feature values due to large magnitudes. The normalized feature vectors are then transformed through fully connected layers, where a 64-neuron dense layer introduces non-linearity, allowing for more complex feature interactions. Batch normalization is used to stabilize learning by standardizing feature distributions and improving gradient flow, leading to faster convergence and better generalization. This entire process for one feature is shown by the four blocks in each row in Figure 5. Instead of treating each extracted feature set independently, the model employs a fusion mechanism where feature representations from all three levels are concatenated into a unified feature descriptor (the white block in Figure 5). This approach integrates information across different abstraction levels, allowing the model to make more informed classification decisions. The fused feature vector undergoes further transformation through a dense layer with 16 neurons, refining the representation before classification. To prevent overfitting, a dropout layer with a probability of 0.45 is applied at this stage, randomly deactivating neurons during training and ensuring that the model does not rely on specific patterns that may not generalize well.

The final classification layer consists of a softmax activation function with two neurons, representing the benign and malignant classes. This output layer assigns a probability score to each class, allowing for precise decision-making in binary classification tasks. By leveraging deep feature extraction, multi-scale fusion, and regularization techniques, the proposed model significantly improves the accuracy and robustness of breast cancer classification in histopathology images compared to the previous two models, as well as objectively. Figure 5 diagrammatically describes the flow of the updated CNN architecture (see Table 5).

**Table 5:** Hyperparameters for the final binary classification model.

Hyperparameter	Value	Explanation
Base model	DenseNet121 (pre-trained on ImageNet)	Acts as the backbone for feature extraction, leveraging pre-trained hierarchical features.
Input image size	(128, 128, 3)	Defines the input shape of images with three color channels (RGB), ensuring consistency across the dataset.
Feature extraction layers	conv3_block12_concat, conv4_block24_concat, conv5_block16_concat	Extracts multi-scale features from different levels of abstraction within the DenseNet121 model.
Pooling method	Global average pooling (GAP)	Reduces dimensionality while retaining essential spatial information from feature maps.
Feature normalization	L2 normalization	Ensures all extracted features contribute proportionally, preventing large-magnitude features from dominating learning.
Hidden dense layer (feature processing)	64 neurons, ReLU activation, L2 regularization (0.001)	Introduces non-linearity to enhance feature interactions and prevents overfitting via L2 regularization.
Batch normalization	Applied after dense layers	Standardizes feature distributions, improving gradient flow and training stability.
Feature fusion	Concatenation of three feature levels	Integrates low-level, mid-level, and high-level features into a unified representation for better classification.
Final dense layer	16 neurons, ReLU activation, L2 regularization (0.001)	Further refines the fused feature representation before classification.
Dropout rate	0.45	Randomly deactivated neurons to prevent overfitting, ensuring better generalization.
Output layer	2 neurons, softmax activation	Generates class probabilities for binary classification (Benign vs. Malignant).
Loss function	Categorical cross-entropy	Suitable for multi-class classification tasks, even though the output is binary (ensures numerical stability).
Optimizer	Adam (learning rate: 0.0001)	Adaptive optimization algorithm that adjusts learning rates dynamically for efficient training.
Learning rate scheduler	ReduceLROnPlateau (factor = 0.5, patience = 3, min_lr = 1e-6)	Reduces the learning rate when validation loss stagnates, helping fine-tune the model.
EarlyStopping	Patience = 5, restore_best_weights = true	Stops training when validation loss stops improving, preventing unnecessary computation and overfitting.
Epochs	50	Maximum number of iterations through the dataset, ensuring the model learns enough before early stopping.

#### 2.2.2 Classifying images as subtypes of benign and malignant

There are four histologically distinct types of benign breast tumors: adenosis (A), fibroadenoma (F), PT, and TA; and four malignant tumors (breast cancer): carcinoma (DC), LC, mucinous carcinoma (MC) and PC.

Adenosis (A) is characterized by the proliferation of glandular structures, which may appear as distorted acini or ducts in biopsy images. These lesions typically show increased glandular tissue without significant atypia, making them relatively easy to differentiate from malignancies under a microscope (Jiang et al., 2017). Fibroadenomas (F) are solid tumors composed of both glandular and stromal components, presenting as well-circumscribed masses with minimal cellular atypia. Their typical pattern in biopsy images shows a lobular architecture with a fibrous stroma, which contrasts with the irregular structures seen in malignant lesions (Schulz-Wendtland et al., 2016). PTs, though benign in some cases, can show features suggestive of malignancy, such as rapid growth and hypercellularity. Their biopsy images typically display leaf-like structures, hence the name, with prominent stromal overgrowth and areas of necrosis that can challenge differentiation from malignant tumors (Barker et al., 2018). TAs are another benign subtype characterized by well-formed tubules lined by a single layer of epithelial cells. These lesions often have minimal cellular atypia, and their biopsy images demonstrate small, uniformly sized tubules with little stroma (Pike et al., 2020).

In contrast, malignant breast cancers are often more challenging to classify due to the diversity of their histopathological features. DC is the most common type of breast cancer and typically appears as irregularly shaped masses with infiltrating ductal structures. The biopsy images show marked cellular pleomorphism, nuclear atypia, and often necrotic areas, which are indicative of aggressive behavior (Tung et al., 2016). LC presents with a distinctive pattern in biopsy images, with tumor cells growing in a single-file pattern and a lack of cohesive cell groups. This “Indian file” arrangement of cells and the absence of desmoplastic stroma are key features that differentiate it from DC (Vargas et al., 2017). Mucinous carcinoma (MC) is characterized by the production of extracellular mucin, which can be observed in the biopsy images as abundant mucin pools, separating the tumor cells. The cells in mucinous carcinoma are typically round with mild pleomorphism and are surrounded by mucin-rich stroma (Manning et al., 2018). Finally, PC shows prominent papillary structures with fibrovascular cores, surrounded by atypical epithelial cells. The tumor cells are often arranged in well-defined papillae, and the biopsy images reveal a complex architecture with cystic spaces and dense fibrous stroma (Thompson et al., 2019).

A CNN was initially trained to distinguish breast cancer subtypes by learning hierarchical spatial features from biopsy images. It captured architectural differences such as the regular tubules in TAs and the disorganized ductal structures in carcinomas, as well as key histological features like pleomorphism, stromal composition, and mucin presence (Rajendran et al., 2018; Srinivasu et al., 2020). The architecture included three Conv2D layers (32, 64, 128 filters), ReLU activations, and (3 × 3) kernels for efficient feature extraction, with max pooling and dropout (0.25/0.5) to reduce overfitting. A dense layer with 512 neurons and a softmax output layer enabled multi-class classification, optimized using categorical cross-entropy and various learning rates (Adam 0.0001, SGD 0.01, RMSprop 0.0001). Training was conducted over 30 epochs with a batch size of 32. Despite these efforts, the model struggled with generalization and subtype discrimination, yielding poor validation performance. Following the success of the DenseNet-based binary model, the architecture was extended and adapted to a multiclass setting, as detailed in Section 2.3 (see Table 6).

**Table 6:** Hyperparameters for the initial subclass classification model.

Hyperparameter	Value	Explanation
Input image size	(256, 256)	The spatial dimensions of each input image are fed to the model.
Batch size	32	Number of images processed at once during training.
Learning rate (Adam)	0.0001	Speed at which the model updates weights during training using Adam optimizer.
Learning rate (SGD)	0.01	Speed of weight updates for the SGD optimizer.
SGD momentum	0.9	Momentum helps accelerate SGD by dampening oscillations during updates.
Learning rate (RMSprop)	0.0001	Step size for weight updates using RMSprop optimizer.
Conv2D filters (1st layer)	32	Number of filters in the first convolutional layer.
Conv2D filters (2nd layer)	64	Number of filters in the second convolutional layer.
Conv2D filters (3rd layer)	128	Number of filters in the third convolutional layer.
Kernel Size	(3, 3)	Size of the sliding window applied in convolution operations.
Activation function	ReLU	Non-linear function applied to neuron outputs to introduce non-linearity.
Dropout rate (conv layers)	0.25	The fraction of neurons dropped to prevent overfitting during training.
Dropout rate (dense layer)	0.5	Fraction of dense layer neurons dropped for regularization.
pooling size	(2, 2)	Size of the window for max pooling operation to reduce feature map dimensions.
Dense layer size	512	Number of neurons in the fully connected layer before the output.
Output layer activation	Softmax	Ensures output values represent probabilities for multi-class classification.
Loss function	Categorical cross-entropy	Measures model error for multi-class classification tasks.
Number of epochs	30	Total number of complete passes through the training dataset.

To enhance diagnostic granularity, we developed two separate CNN classifiers—one for benign subtypes and another for malignant subtypes—rather than training a single multi-class model across all categories to extend the binary class classifier to multiple classes. This decision is rooted in both clinical and computational rationale. Clinically, benign and malignant lesions differ not just in severity but in their underlying histomorphological characteristics, tissue organization, and staining patterns (Barker et al., 2018; Pike et al., 2020). Combining both under one classifier risks underrepresenting these distinct patterns during training, especially when using class-imbalanced datasets like BreakHis, which contain fewer samples for certain subtypes. Computationally, separate models allow more focused feature learning within each class family, minimizing intra-class confusion. Prior studies have shown that domain-specific subnetworks often outperform unified multi-class models in pathology, especially when classes exhibit heterogeneity in structure and frequency (Gandomkar et al., 2018; Patel et al., 2024). For example, Umer et al. (2022) observed that a specialized multi-branch CNN improved the classification of individual breast cancer subtypes, while Arevalo et al. (2016) showed that focused lesion-specific training led to better mass detection in mammography. Furthermore, multi-class CNNs trained across all subtypes often suffer from performance trade-offs—gaining sensitivity in one category while losing specificity in others (McKinney et al., 2020). By using separate networks, we achieved higher per-class precision and recall, especially for visually similar classes like fibroadenoma and PT. This modular architecture also supports more targeted clinical applications, such as malignancy-specific triaging or subtype-based treatment suggestion systems, which align with current trends in personalized digital pathology (Lee et al., 2024).

The multi-class CNN architectures for subtype classification were directly adapted from the binary classification model described previously. DenseNet121, used as the backbone in both approaches, provides efficient deep feature extraction through its dense connectivity and hierarchical learning capabilities. As in the binary model, features were extracted from three depths—conv3_block12_concat, conv4_block24_concat, and conv5_block16_concat—capturing progressively abstract morphological features necessary for histopathological differentiation. These include low-level structures such as cellular edges and textures, mid-level patterns like glandular and stromal organization, and high-level attributes such as nuclear pleomorphism and mitotic activity.

Each extracted feature map underwent GAP, L2 normalization, and transformation *via* a dense layer with L2 regularization, batch normalization, and dropout, following the same processing pipeline outlined in the binary classifier. The processed features were then concatenated into a unified multi-scale descriptor and passed through a final dense layer before classification.

Instead of a binary output, the final softmax layer consisted of four neurons to predict between the benign or malignant subtypes, depending on the network. Two separate CNNs were trained—one for benign subtypes and another for malignant—to reduce feature entanglement and focus each model on intra-class variation. This design choice improved the model’s capacity to learn subtle distinctions specific to each class group, such as distinguishing fibroadenoma from PT, or DC from PC.

**Figure 6:**
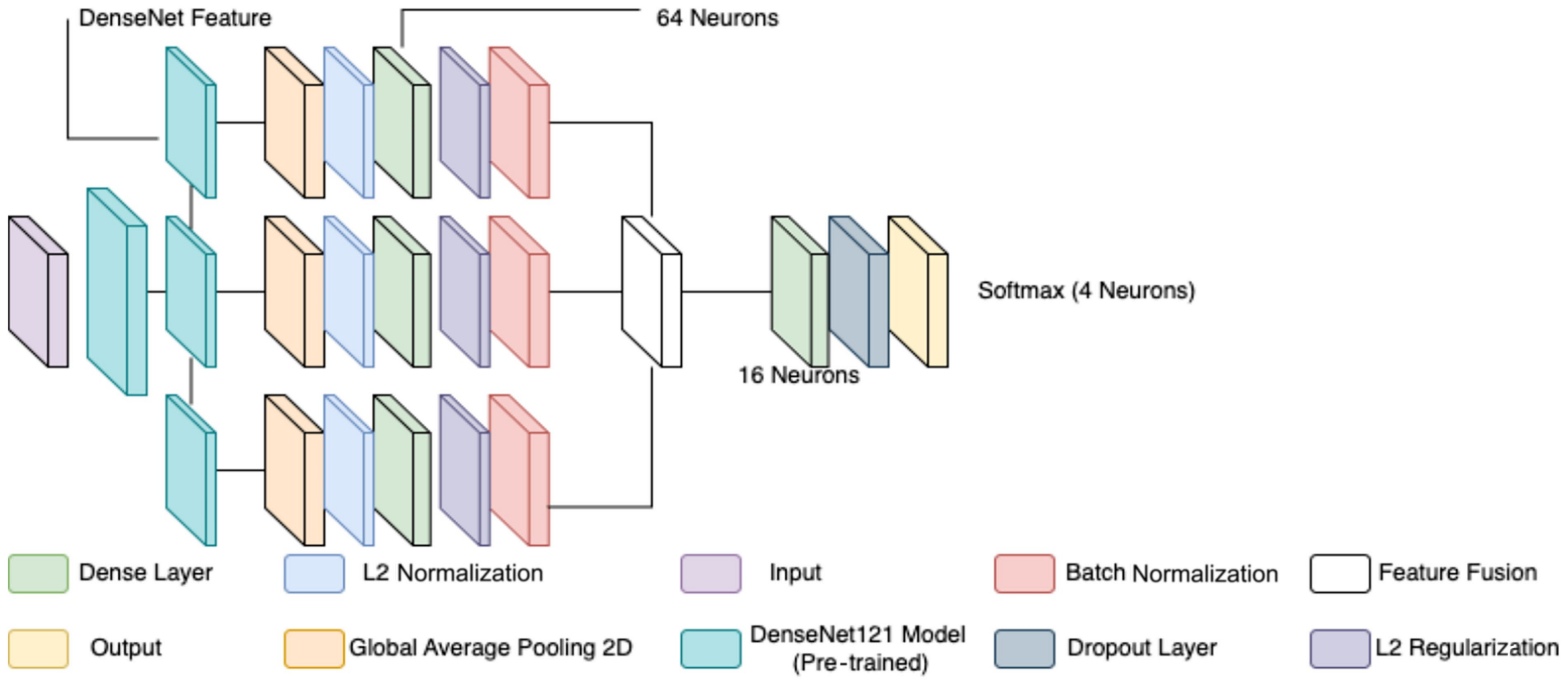
CNN used for multi-class classification.

**Figure 7:**
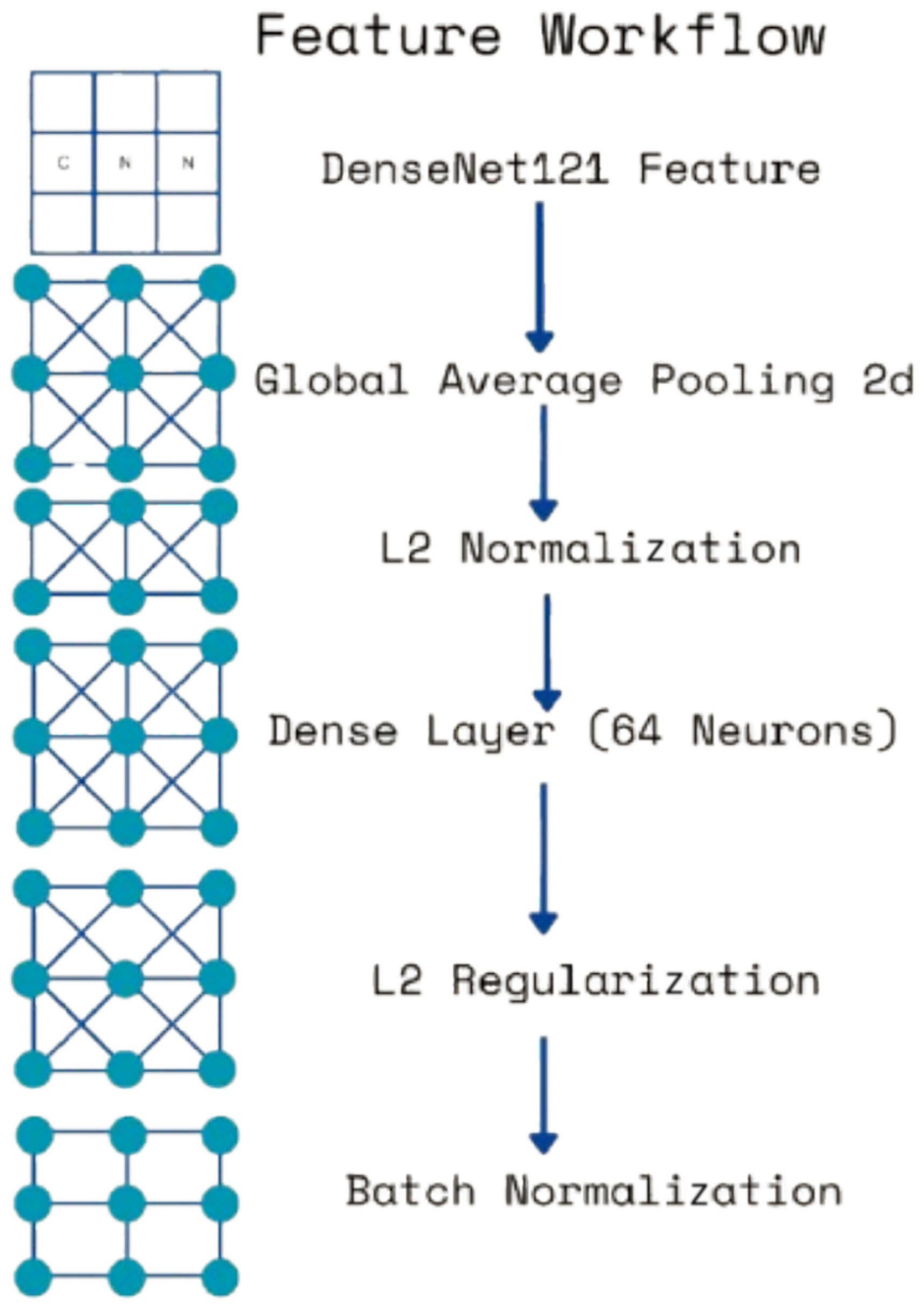
Diagram for feature workflows.

Optimization and training procedures mirrored those of the binary classification model, utilizing the Adam optimizer (learning rate 0.0001), EarlyStopping, and ReduceLROnPlateau for improved convergence and generalization (see Figures 6, 7 and Table 7).

**Table 7:** Hyperparameters for the final subclass classification model.

Hyperparameter	Value	Explanation
Base model	DenseNet121 (pre-trained on ImageNet)	Acts as a deep feature extractor, leveraging hierarchical feature learning for efficient classification.
Input image size	(128, 128, 3)	Ensures computational efficiency while preserving essential histopathological details. A higher resolution, like 256 × 256, would increase memory usage without significantly improving feature representation.
Feature extraction layers	conv3_block12_concat, conv4_block24_concat, conv5_block16_concat	Extracts features at multiple abstraction levels, capturing both fine-grained and high-level morphological details.
Pooling method	Global average pooling (GAP)	Reduces dimensionality while preserving spatial characteristics of the extracted feature maps.
Feature normalization	L2 normalization	Prevents feature values with high magnitudes from dominating the learning process, ensuring numerical stability.
Hidden dense layer (feature processing)	64 neurons, ReLU activation, L2 regularization (0.001)	Introduces non-linearity for complex feature interactions and applies L2 regularization to prevent overfitting.
Batch normalization	Applied after dense layer s	Standardizes feature distributions, accelerating convergence and improving training stability.
Feature fusion	Concatenation of three feature levels	Integrates information across different levels of abstraction, enhancing classification performance.
Final dense layer	16 neurons, ReLU activation, L2 regularization (0.001)	Refines the multi-scale feature representation before classification.
Dropout rate	0.45	Prevents overfitting by randomly deactivating neurons, ensuring better generalization.
Output layer	4 neurons, softmax activation	Outputs probability scores for the four benign subtypes.
Loss function	Categorical cross-entropy	Suitable for multi-class classification tasks, optimizing probability-based learning.
Optimizer	Adam (learning rate: 0.0001)	Adaptive optimization that adjusts learning rates dynamically for efficient and stable training.
Learning rate scheduler	ReduceLROnPlateau (factor = 0.5, patience = 3, min_lr = 1e-6)	Reduces learning rate when validation loss stagnates, improving fine-tuning of weights.
EarlyStopping	Patience = 5, restore_best_weights = true	Stops training when validation loss stops improving, preventing unnecessary computation and overfitting.
Epochs	50	Maximum number of training iterations through the dataset, ensuring sufficient learning.

### 2.3 Novel characteristics of the model

The proposed classification algorithm is distinguished by its multi-scale feature fusion architecture built on DenseNet121. Instead of relying only on the final layer features (as done in most standard CNN classifiers), it extracts and fuses features from three different depths of the DenseNet (the end of conv3, conv4, and conv5 blocks). Each feature map is subjected to GAP and L2 normalization, then passed through a small fully connected layer with batch normalization (and dropout) before concatenation. This design enables the model to capture discriminative patterns at multiple spatial scales—from fine-grained cellular details to higher-level tissue organization—within a single network. In essence, the model behaves like an integrated multi-scale ensemble, where lower-level texture features and higher-level semantic features jointly inform the final prediction. This multi-scale fusion is particularly well-suited to histopathology images, where diagnostically relevant cues appear at different magnifications. Prior work has recognized the value of multi-scale information: Araújo et al. (2017) designed a custom CNN to explicitly capture both nuclei-level details and global tissue architecture (Araújo et al., 2017), and Gupta and Bhavsar (2018) similarly argued that “different layers… contain useful discriminative information” for classifying breast histology images (Gupta and Bhavsar, 2018). The proposed model builds on this principle by simultaneously leveraging DenseNet’s intermediate feature maps, rather than using only the deepest features or requiring multiple separate networks.

By fusing intermediate feature vectors, our approach yields a richer representation that combines context from multiple receptive field sizes. This strategy is analogous to the “hypercolumn” or feature pyramid concept—effectively consolidating features at various levels of abstraction. In histopathology, such multi-level fusion has clear advantages: for example, benign vs. malignant differentiation may depend on both cellular morphology and the broader tissue architecture. Traditional transfer-learning classifiers often discard these multi-level cues by only using the final global feature map of a pre-trained CNN. In contrast, our DenseNet121-based model preserves multi-scale feature information by tapping conv3, conv4, and conv5 outputs. Zhu et al. (2019) demonstrated a similar idea with a two-branch “global vs. local” CNN ensemble, where merging a whole-image branch with a high-zoom patch branch improved representation power (Zhu et al., 2019). Our method achieves a comparable multi-scale effect within a single backbone: the conv3_block output (earlier layer) can focus on local textural patterns (analogous to a high-magnification view), while the conv5_block output provides global context (analogous to a low-magnification overview). The DenseNet architecture’s dense connectivity may further facilitate this feature reuse across scales (Huang et al., 2017). Notably, DenseNet121 has been a popular choice in medical image analysis due to its efficient feature propagation; several breast histopathology studies report strong results with DenseNet-based transfer learning—for example, Liew et al. (2021) achieved ~97% accuracy by using DenseNet201 features with an XGBoost classifier (Liew et al., 2021). Our model differentiates itself by not only fine-tuning DenseNet121 on the task, but by modifying its topology to combine multi-scale features—a design rarely explored in prior breast cancer studies.

Another novel aspect of our approach is the use of L2 normalization and balanced regularization in the feature fusion process. Each branch’s pooled feature vector is L2-normalized before fusion, ensuring that no single scale’s features dominate the others due to scale differences. This is an uncommon yet effective practice in classification networks—more frequently seen in metric learning or multi-instance aggregation contexts—and it promotes stability when training the fused classifier. For instance, Xie et al. (2020) applied L2 normalization to patch feature vectors in a histopathology model to stabilize feature magnitudes (Xie et al., 2020). In our design, the L2 norm acts similarly to a scale calibration across the three feature streams, so that the subsequent dense layers operate on features of comparable norm. We then apply dropout and batch normalization in each branch’s dense layer (as well as the final classification layer) to combat overfitting. High-resolution histology images are prone to overfitting given their limited datasets, and regularization is critical. Previous works have indeed found that naive CNN models can overfit histopathology data even with dropout—for example, a shallow CNN still overfit BreakHis patches even with 20% dropout, as reported by Kode and Barkana (2023) (Kode and Barkana, 2023). By combining dropout with batch normalization and the inherent regularization of feature fusion, our model aims to generalize better. The inclusion of batch normalization after the small dense layers also helps each scale-specific feature vector to be well-conditioned before merging, which eases the joint learning. These design choices—L2 normalization of features and dropout-regularized dense layers for each scale—are unique refinements that distinguish our architecture from standard fine-tuned CNN classifiers that often use only a global pooling and a linear classifier on top.

Compared to existing deep learning methods in breast cancer histopathology, the proposed model offers a new balance of simplicity and multi-scale sophistication. Many prior studies have focused either on transfer learning with single CNNs or on ensembles of multiple models. On the one hand, simple transfer-learning approaches, fine-tuning a single network (e.g., ResNet or DenseNet), have shown strong baseline performance (Spanhol et al., 2016; Wahab et al., 2017), but they might miss multi-scale cues. Some recent works augment such models with attention mechanisms—for example, an EfficientNetV2 with a channel-spatial attention module (CBAM) by Aldakhil et al. (2024) outperformed standard CNNs, reaching ~92–94% accuracy on BreakHis magnifications (Aldakhil et al., 2024). Others integrate vision transformers to capture global context—for example, Mehta et al. (2022) introduced a ViT-based HATNet that achieved state-of-the-art accuracy (Mehta et al., 2022). However, these sophisticated models often remain single-scale in the sense that they ultimately rely on one level of feature map (global features) enhanced via attention. On the other hand, ensemble and multi-branch strategies explicitly leverage multi-scale inputs. For example, Umer et al. (2022) proposed *6B-Net*, a six-branch CNN with different receptive field sizes in each branch, and fused their outputs for classifying the eight BreakHis classes (Umer et al., 2022). Similarly, an earlier study by Zhu et al. (2019) assembled multiple compact CNNs (and even pruned channels) to combine local and global predictions for histology images (Zhu et al., 2019). These methods confirmed that combining features from multiple scales or multiple models can boost accuracy. Our approach achieves this multi-scale fusion within a single pre-trained DenseNet, rather than requiring separate networks for different magnifications or an external ensemble of classifiers. This provides a more unified and computationally efficient framework: for instance, Wakili et al. (2022) reported a DenseNet-based transfer model (“DenTnet”) that attained ~99% accuracy on BreakHis (Wakili et al., 2022), but that model treats DenseNet as a black-box feature extractor for an SVM or softmax classifier. In contrast, our model integrates the multi-level feature extraction into the training process, which could offer better synergy between feature learning and classification. It also simplifies the pipeline—there is no need for post-CNN classifiers like SVM (as used by Araújo et al., 2017 and others) or gradient boosting ensembles (as in Liew et al., 2021) because the fused deep features are learned end-to-end to directly optimize classification.

In summary, the novelty of our algorithm lies in combining multi-scale feature fusion, transfer learning with DenseNet121, and strategic normalization/regularization into one coherent model for breast histopathology classification. This design captures the multi-scale nature of tissue patterns more explicitly than standard CNN classifiers. It leverages DenseNet’s strength in feature reuse while addressing multiple scales akin to multi-branch networks, but with fewer parameters and a single-pass inference. By comparing with the literature, we see that our approach is unique in *how* it fuses intermediate CNN features: Gupta and Bhavsar (2018) sequentially processed DenseNet layers’ outputs rather than fusing them, and most other DenseNet-based methods (Wakili et al., 2022; Liew et al., 2021) used only the final layer or combined whole-network outputs in ensembles. Moreover, the inclusion of L2-normalized feature vectors and dropout-regularized dense layers for each scale is a novel architectural choice that, to our knowledge, has not been explicitly reported in prior breast cancer histopathology studies. These innovations position our model as a multi-scale, multi-level classifier that is well-aligned with the visual hierarchy a pathologist employs (from cells to tissue architecture), setting it apart from conventional single-scale CNN models and even from recent attention-based or ensemble-based state-of-the-art methods in this domain.

### 2.4 Metrics evaluations

In AI, especially in domains like classification and regression, evaluation metrics play a vital role in measuring model performance. Within medical diagnostics, metrics such as precision and recall are particularly significant. Precision reflects the proportion of correct positive predictions, meaning that when a model identifies cancer, it is likely to be correct—thereby reducing unnecessary alarm (Sokolova and Lapalme, 2009). Recall, on the other hand, focuses on identifying as many actual positive cases as possible, which is crucial in healthcare settings to avoid overlooking genuine cases of disease (Sokolova and Lapalme, 2009). Although accuracy is a common metric indicating the percentage of correctly predicted instances overall, it can be deceptive in scenarios with class imbalance, as it does not account for the nature of errors made (Tharwat, 2020).

To address such limitations, the F1-score is often employed. This metric, calculated as the harmonic mean of precision and recall, offers a more balanced view by considering both false positives and false negatives—an essential factor in medical imaging, where failing to detect malignant tumors can have serious consequences (Chicco and Jurman, 2020). Additionally, the area under the curve (AUC) of the receiver operating characteristic (ROC) curve is another key metric. It evaluates the model’s capacity to distinguish between positive (cancerous) and negative (non-cancerous) cases, with scores approaching 1 indicating high discriminative ability (Yang and Ying, 2022). Given the frequent imbalance in medical datasets, relying on a combination of these metrics provides a more reliable and holistic evaluation of model effectiveness (see Table 8).

**Table 8:** Metrics for evaluation of the algorithm.

Metric	Formula	Explanation	References
Precision	TP/(TP + FP)	Measures the proportion of correctly predicted positive cases out of all predicted positives.	Sokolova and Lapalme (2009)
Recall (sensitivity)	TP/(TP + FN)	Measures the proportion of actual positive cases correctly identified by the model.	Sokolova and Lapalme (2009)
F1-score	2(Precision*Recall)/(Precision+Recall)	Harmonic mean of precision and recall, providing a balanced measure for imbalanced datasets.	Chicco and Jurman (2020)
Accuracy	(TP + TN)/(TP + TN + FP + FN)	Measures overall correctness of the model across all classes.	Tharwat (2020)
Area under the curve (AUC)	Computed from ROC curve	Represents the probability that the model ranks a randomly chosen positive instance higher than a randomly chosen negative one.	Yang and Ying (2022)

TP (True Positives): Correctly predicted positive cases; TN (True Negatives): Correctly predicted negative cases; FP (False Positives): Incorrectly predicted positive cases; FN (False Negatives): Incorrectly predicted negative cases.

## 3 Results and discussion

The following sections present a comprehensive evaluation of the developed AI models across various experimental phases, including binary classification and multi-class subtype detection for breast cancer biopsy images. Detailed performance metrics such as accuracy, precision, recall, F1-score, and ROC-AUC are reported to assess the effectiveness and generalizability of each model. The study began by analyzing the outcomes of the initial CNN architecture, followed by performance improvements observed with the final DenseNet121-based model. It then extends this evaluation to multi-class classification tasks, detailing the results for both benign and malignant subtypes. These results collectively demonstrate the diagnostic power and clinical viability of the proposed framework.

### 3.1 The binary categorical classification as benign or malignant

The initial CNN architecture, developed for binary classification of histopathology images into benign and malignant categories, was evaluated using different filter and kernel size configurations. Table 9 presented in this section summarizes the performance metrics for each configuration. While the model achieved a maximum accuracy of 84%, this result was below the expected benchmark for binary classification tasks, where an accuracy of 95% or higher is generally required to maintain robust performance during the transition to multi-class classification.

**Table 9:** Initial results of binary classification.

Filters	Mesh size	Precision	Recall	Binary accuracy	AUC
32, 32, 32	3×3	0.8603	0.9283	0.8424	0.8768
16, 32, 16	4×4	0.8893	0.8994	0.8542	0.8872
16, 32, 16	5×5	0.8253	0.8465	0.7799	0.8290

**Figure 8:**
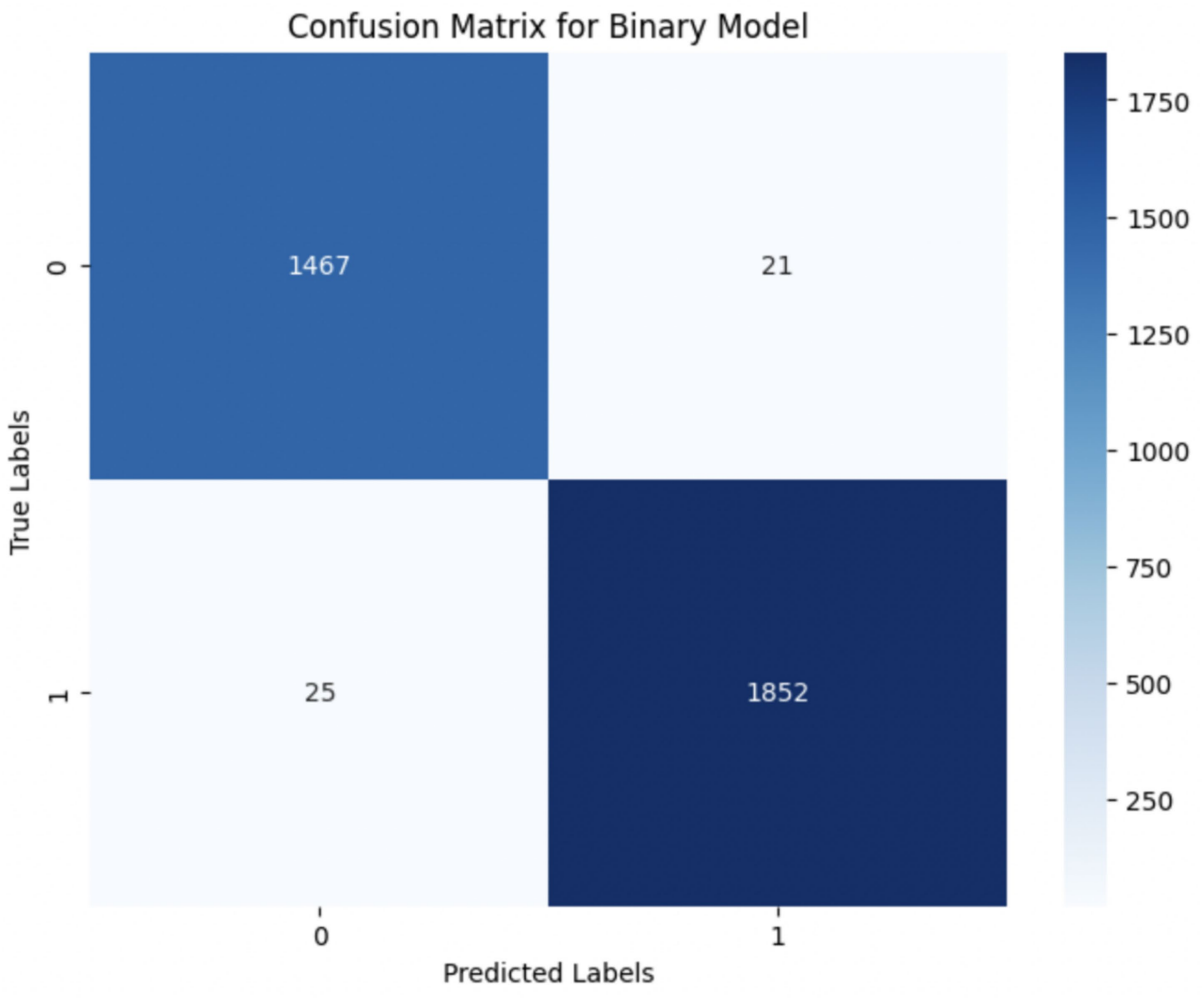
Confusion matrix for final binary classification.

**Figure 9:**
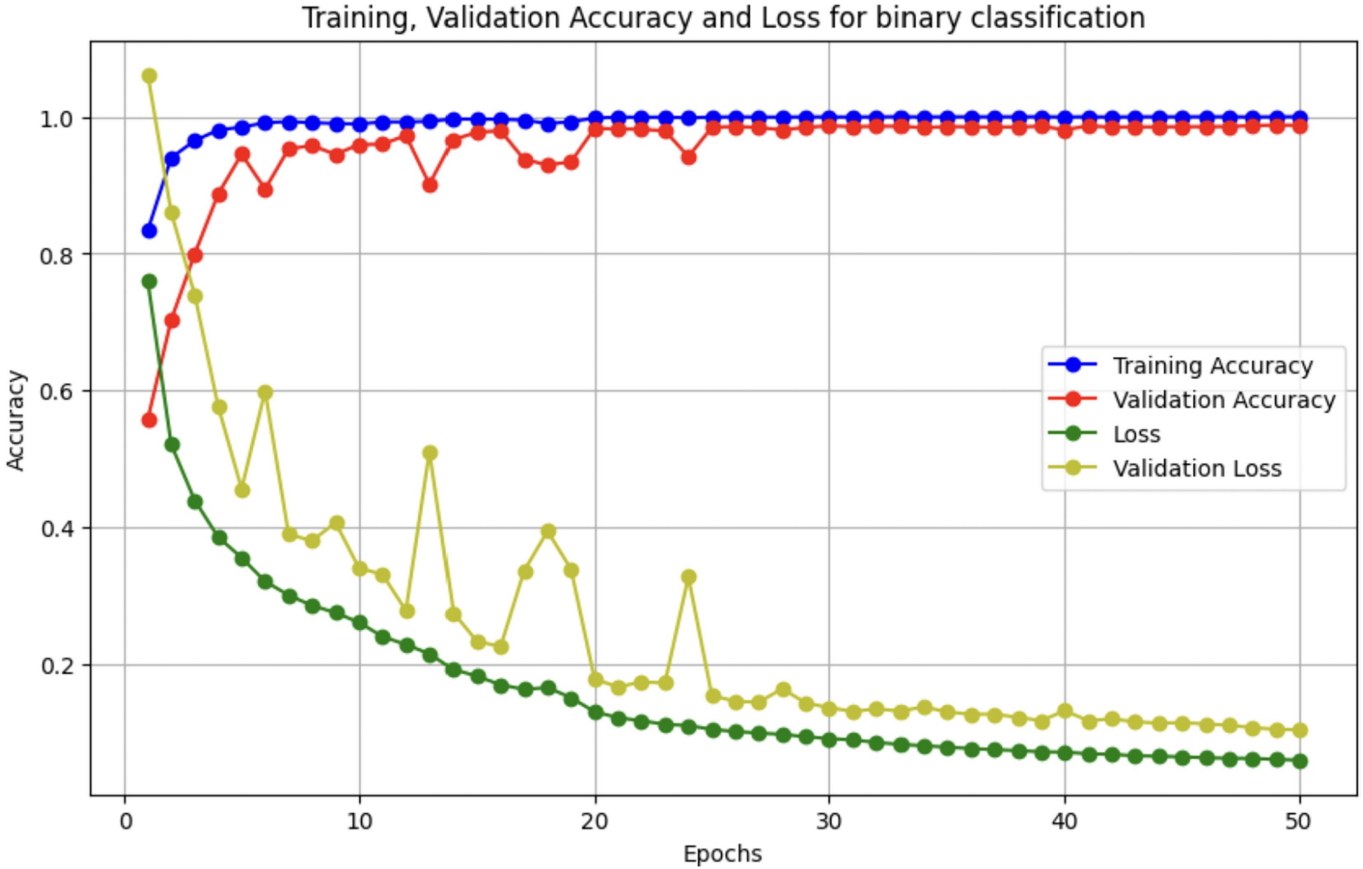
Accuracy/loss vs. epoch graph.

Upon using the proposed DenseNet121-based binary classification, the model demonstrated outstanding performance in distinguishing between benign and malignant breast cancer histopathology images. The model was trained on out-of-sample biopsy images. As shown in Table 10, the model achieved a test accuracy of 98.63%, indicating a high level of reliability in classification. Precision, recall, and F1-score values were also notably high at 0.9888, 0.9867, and 0.9877, respectively, reflecting the model’s ability to correctly identify malignant cases while minimizing false positives and false negatives. The ROC-AUC score of 0.9863 further confirms the model’s strong discriminative capability, showing its robustness in distinguishing between the two classes across different classification thresholds. Figure 8 shows the confusion matrix for the classification, while Figure 9 is a graph showing the change in accuracy and loss with respect to epochs. Compared to previous models that relied on shallow convolutional architectures and dataset fragmentation based on magnification levels, the proposed model benefits from multi-scale feature extraction at different network depths, improving its ability to generalize across varying histopathological patterns. By utilizing a 128 × 128 input image resolution, the model strikes a balance between computational efficiency and feature preservation, allowing for optimal learning without excessive memory consumption. The use of feature fusion across different abstraction levels enhances the classification robustness, ensuring that both low-level structural features and high-level morphological variations contribute to the final decision-making process.

**Table 10:** Final results of binary classification algorithm.

Metric	Value
Test accuracy	0.9863
Precision	0.9888
Recall	0.9867
F1-score	0.9877
ROC–AUC	0.9863

### 3.2 Multi-category classification of benign and malignant cancers

**Figure 10:**
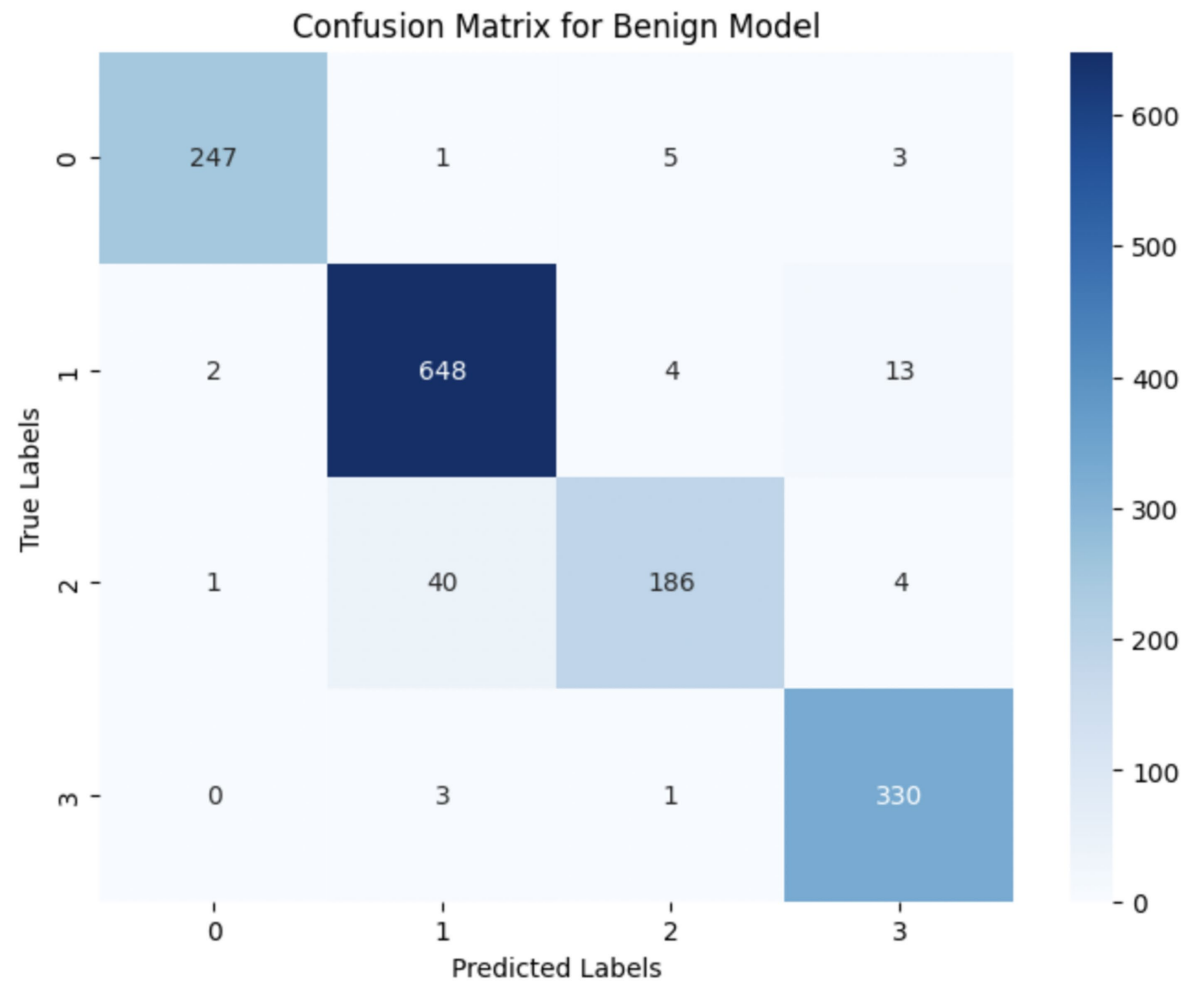
Confusion matrix for benign sub-category classification.

**Figure 11:**
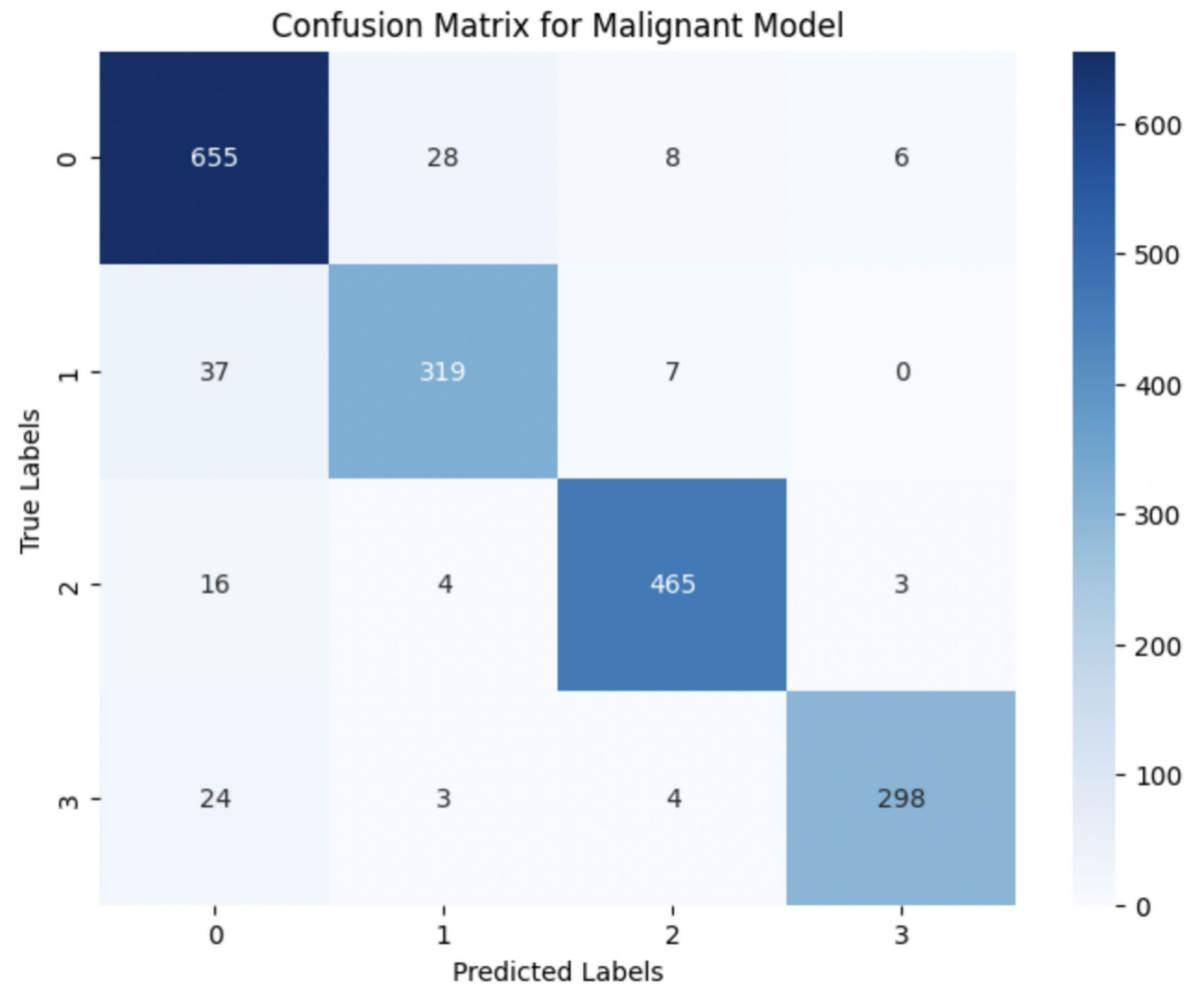
Confusion matrix for malignant sub-category classification.

**Figure 12:**
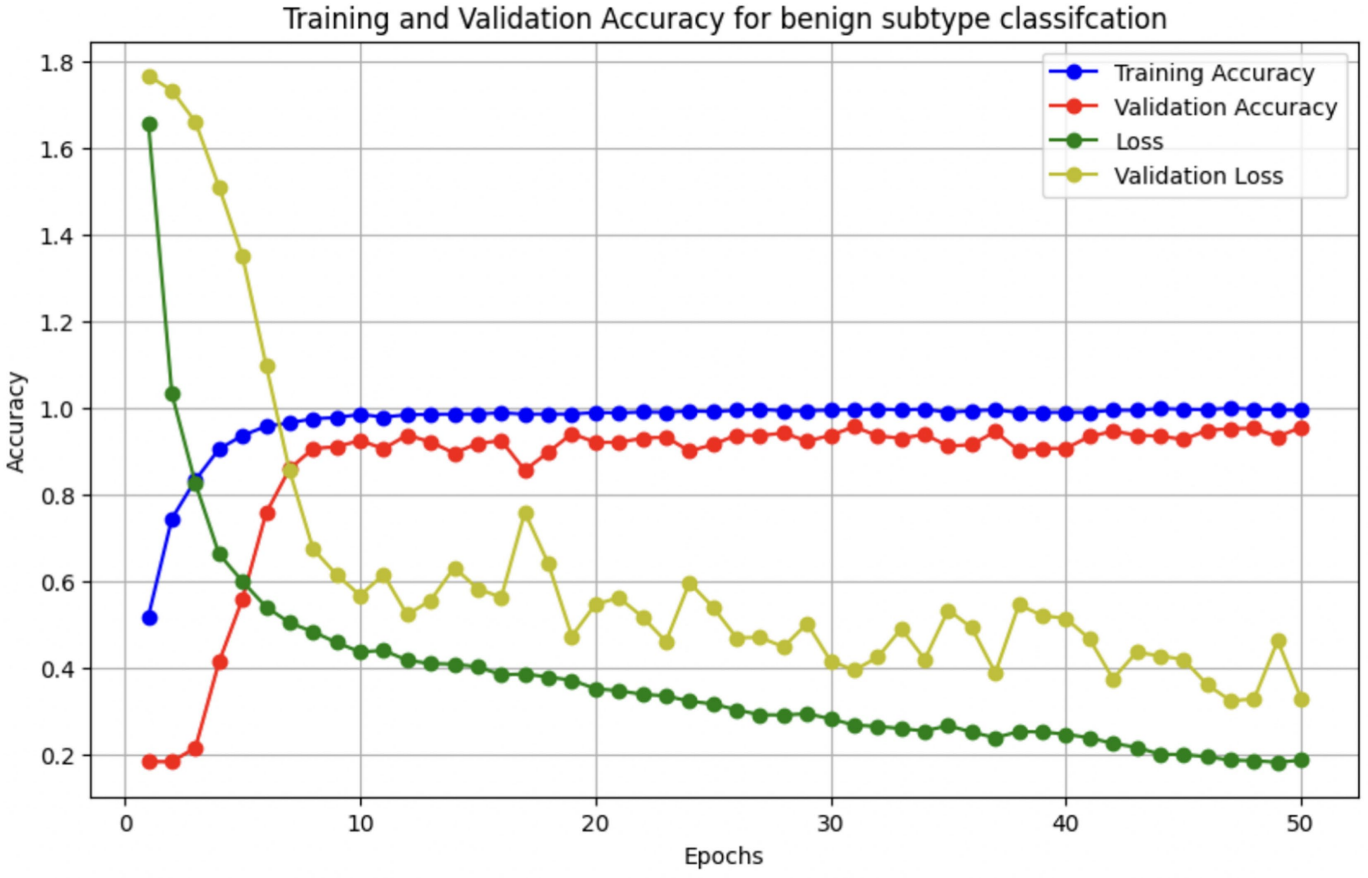
Accuracy/loss vs. epoch graph for benign subclass model.

**Figure 13:**
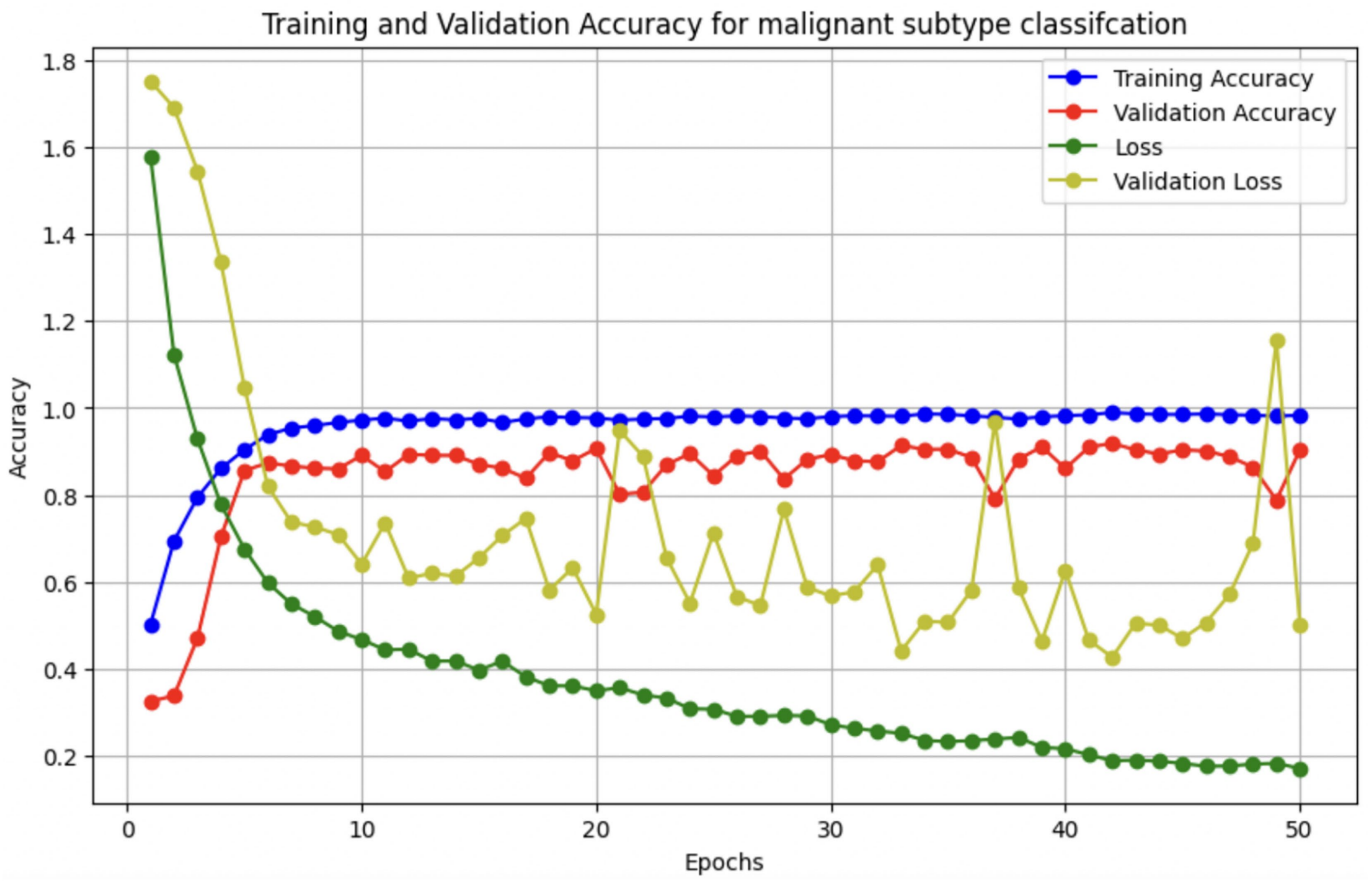
Accuracy/loss vs. epoch graph for malignant subclass model.

The performance of the multi-class classification models for benign and malignant breast cancer subtypes is summarized in Table 11. The model was trained on out-of-sample biopsy images. The model trained for benign classification achieved a test accuracy of 0.9382, whereas the malignant classification model obtained a test accuracy of 0.9158. Additionally, the malignant model demonstrated strong predictive capabilities, with a precision of 0.9151, recall of 0.9240, and an F1-score of 0.9182, while the benign model had a precision of 0.9541, recall of 0.9324 and an F1-score of 0.9415. These results highlight the effectiveness of using separate CNNs for each subtype, allowing for improved feature extraction and discrimination between fine-grained morphological variations. Figure 10 shows the confusion matrix for the benign model, while Figure 11 shows the same for the malignant model. Figure 12 shows the graph of the change in accuracy/loss with respect to the epochs for benign, and Figure 13 shows the same for malignant.

**Table 11:** Final results for multi-category classification.

Classification task	Accuracy	Precision	Recall	F1-score
Benign	0.9483	0.9541	0.9324	0.9415
Malignant	0.9254	0.9318	0.9193	0.9251

The results indicate that the model for benign subtypes outperformed the malignant classification model in terms of overall accuracy. However, the malignant classification model maintained strong recall and F1-score values, demonstrating its ability to correctly identify malignant subtypes while balancing precision. These findings support the hypothesis that training separate models for benign and malignant subtypes allows for more specialized learning, leading to improved classification performance. The integration of DenseNet121 as a feature extractor has further contributed to the model’s ability to capture multi-scale morphological patterns in histopathology images.

#### 3.2.1 Per-class metrics for benign subtype classification

To provide deeper insight into the model’s subclass-level performance, we evaluated per-class precision, recall, and F1-score for the benign tumor categories (Table 12). The model achieved excellent performance for adenosis (F1-score: 0.98) and TA (0.96), both of which exhibited high precision and recall. Fibroadenoma showed a near-perfect recall of 0.99 but a slightly lower precision of 0.89, indicating occasional misclassification of other benign types as fibroadenoma. The most challenging class was PT, with a recall of 0.74 and an F1-score of 0.83, suggesting frequent confusion with other fibroepithelial lesions—particularly fibroadenoma.

**Table 12:** Per-class metrics for benign classes.

Classification task	Precision	Recall	F1-score
Adenosis	0.99	0.96	0.98
Fibroadenoma	0.89	0.99	0.94
Phyllodes tumor	0.94	0.74	0.83
Tubular adenoma	0.98	0.94	0.96

This result aligns with known clinical challenges, as PTs and cellular fibroadenomas often exhibit overlapping histological features, especially under low magnification. Even experienced pathologists can find it difficult to differentiate between these entities, given their shared stromal overgrowth and similar architectural patterns (Barker et al., 2018). As such, this subtype boundary represents a meaningful opportunity for AI to provide diagnostic support. Our findings underscore the importance of refining AI models to handle these borderline cases more effectively.

**Figure 14:**
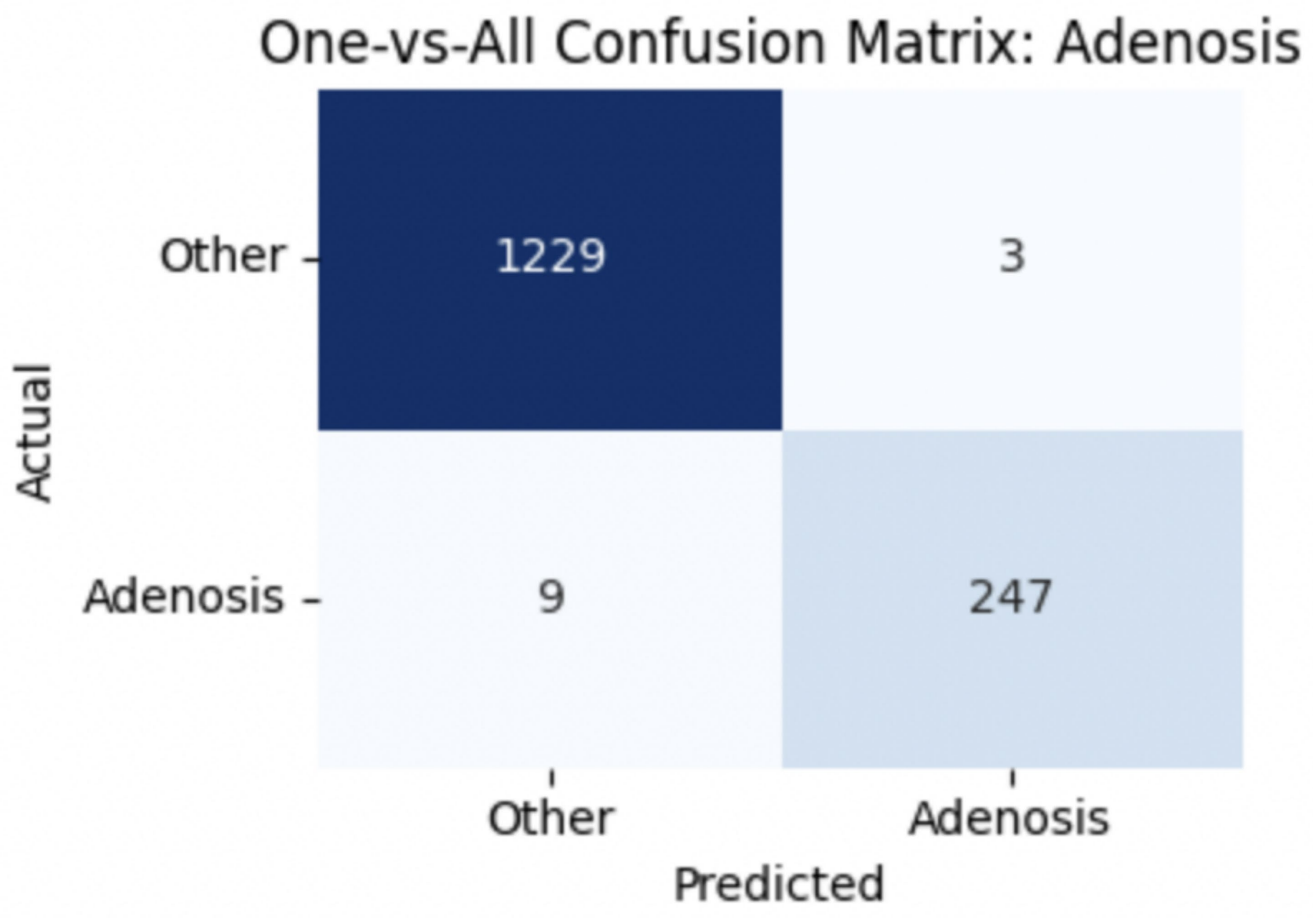
Adenosis confusion matrix.

**Figure 15:**
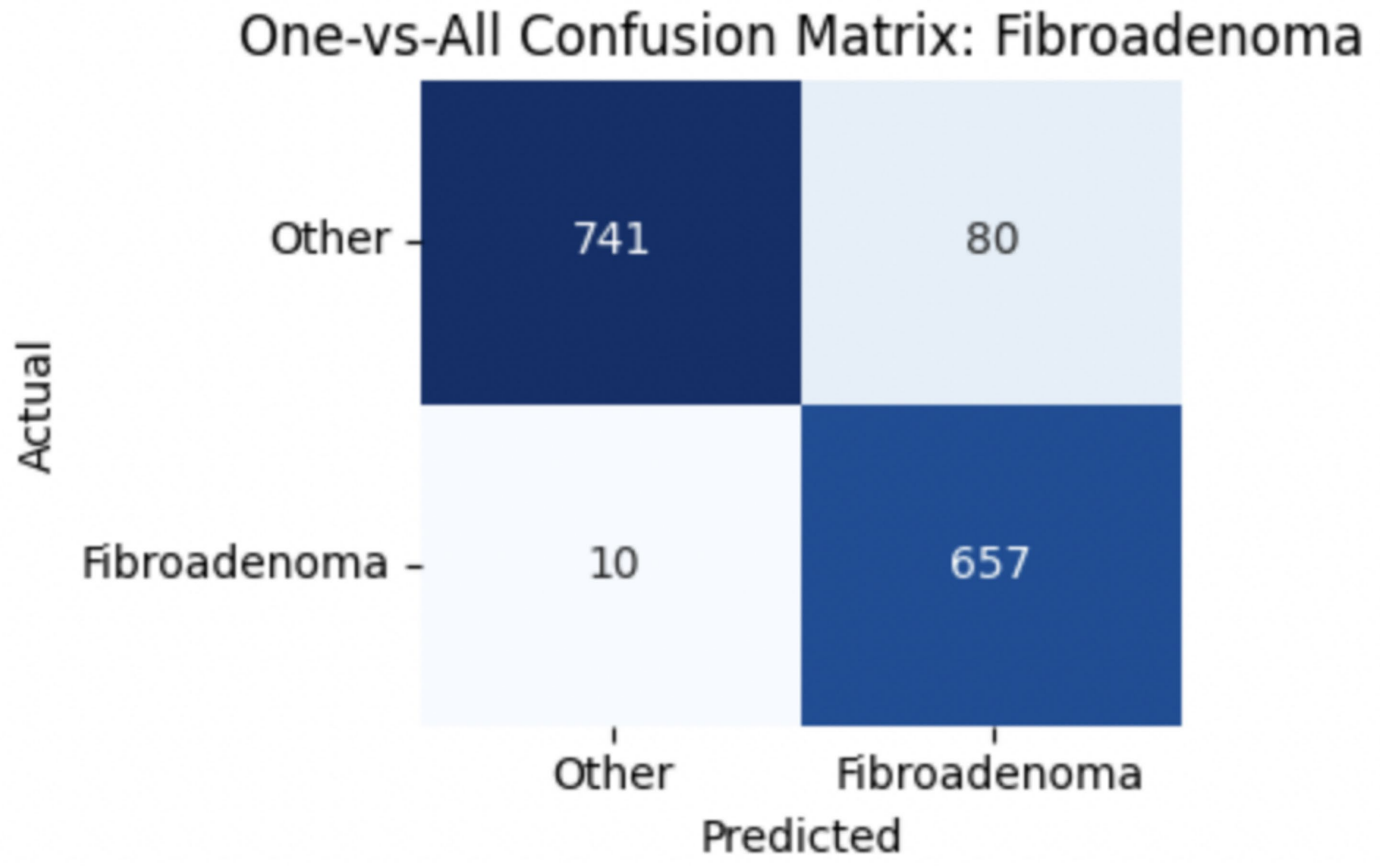
Fibroadenoma confusion matrix.

**Figure 16:**
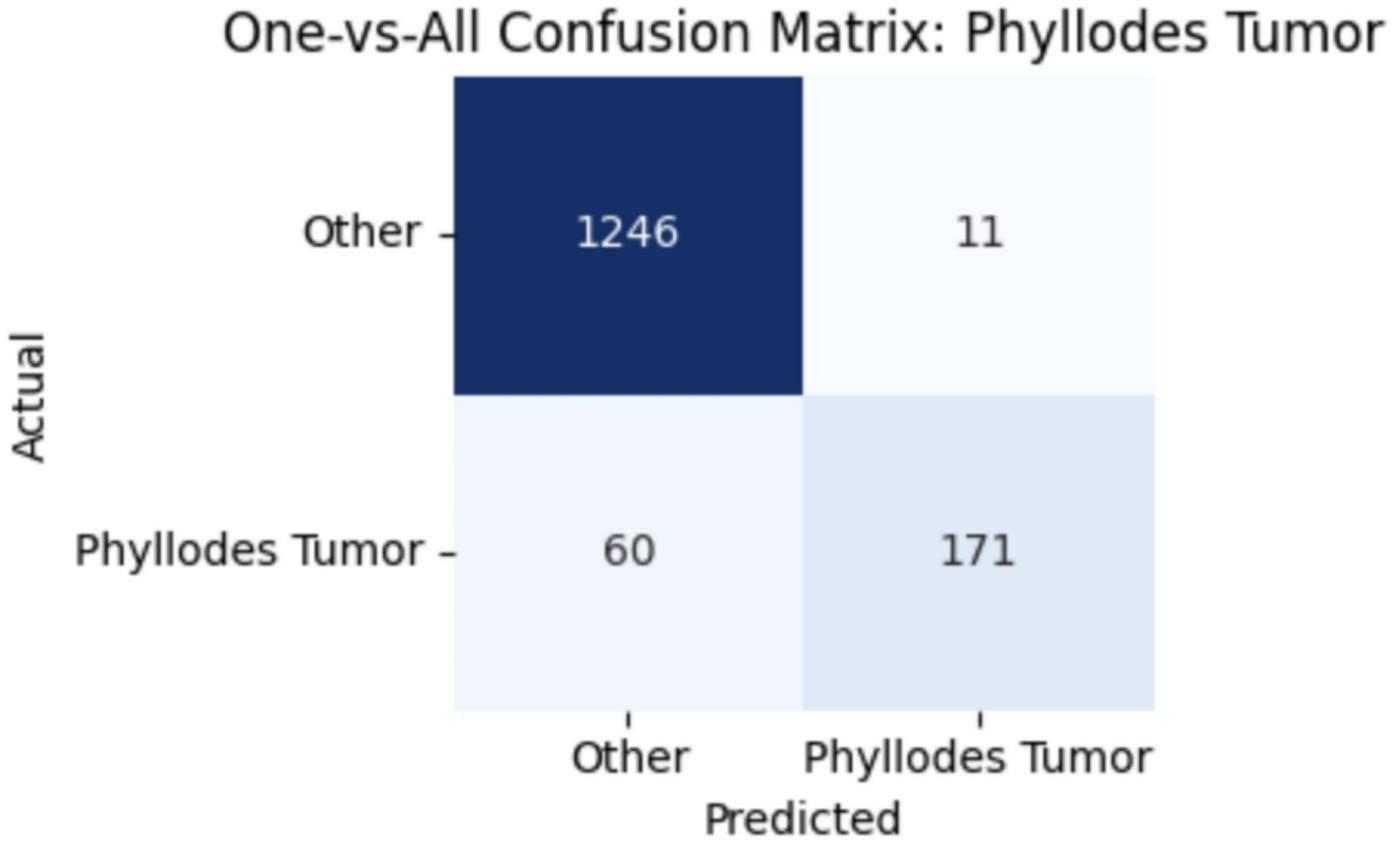
Phyllodes tumour confusion matrix.

**Figure 17:**
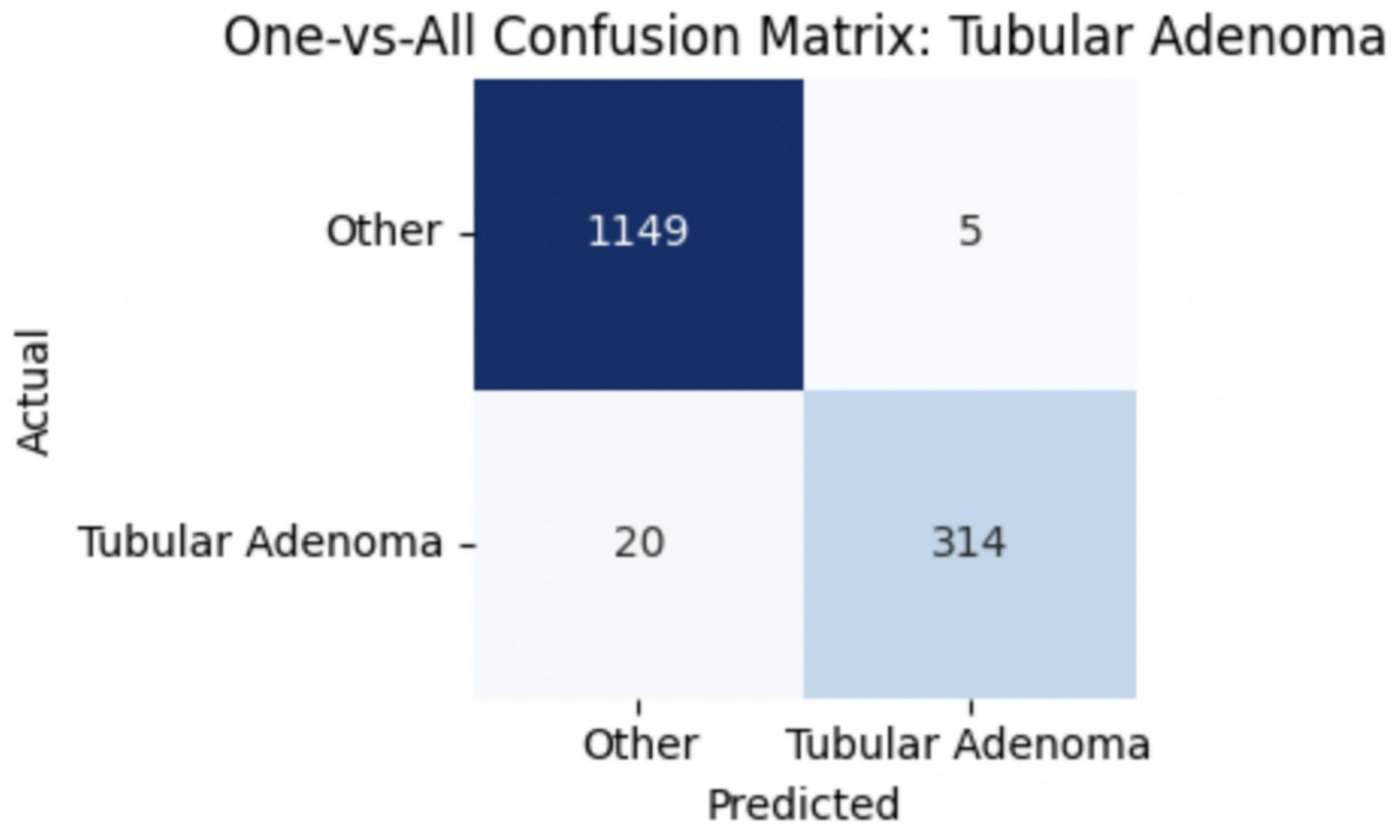
Tubular adenoma confusion matrix.

To improve classification in this region of diagnostic uncertainty, future iterations of the model could incorporate additional histological cues beyond morphology alone—such as mitotic count, stromal cellularity, or margin assessment, which are often critical in distinguishing PTs from fibroadenomas. Enhanced annotation protocols that focus on these differentiating features, particularly at multiple magnifications, could help reduce misclassification. Overall, while the model demonstrates strong performance in benign subtype differentiation, the results also highlight the necessity of targeted refinement in clinically ambiguous classes like PTs. Figures 14–17 show the confusion matrices for Ductal Carcinoma, Lobular Carcinoma, Mucinous Carcinoma, and Papillary Carcinoma, respectively.

#### 3.2.2 Per-class metrics for malignant subtype classification

**Figure 18:**
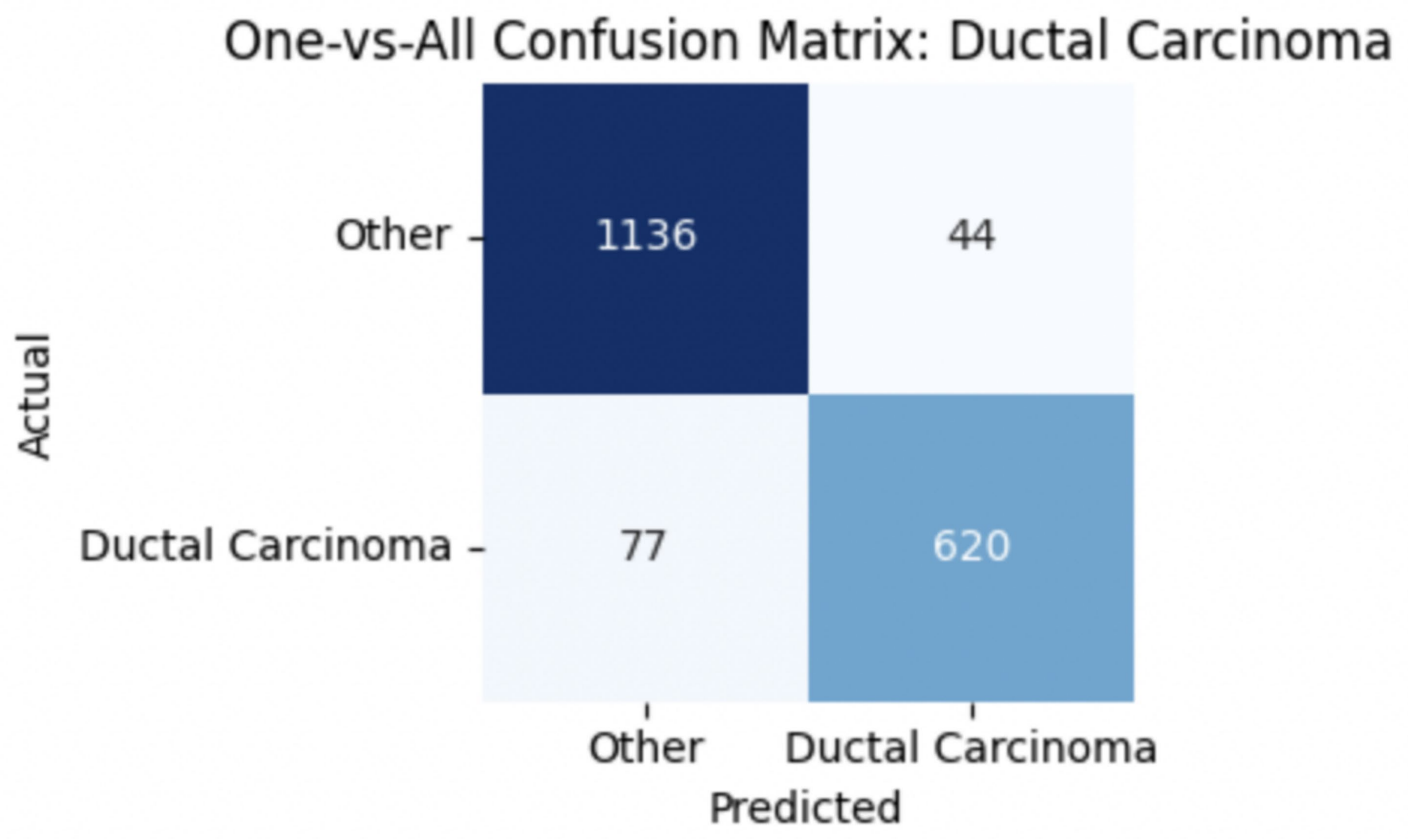
Ductal carcinoma confusion matrix.

**Figure 19:**
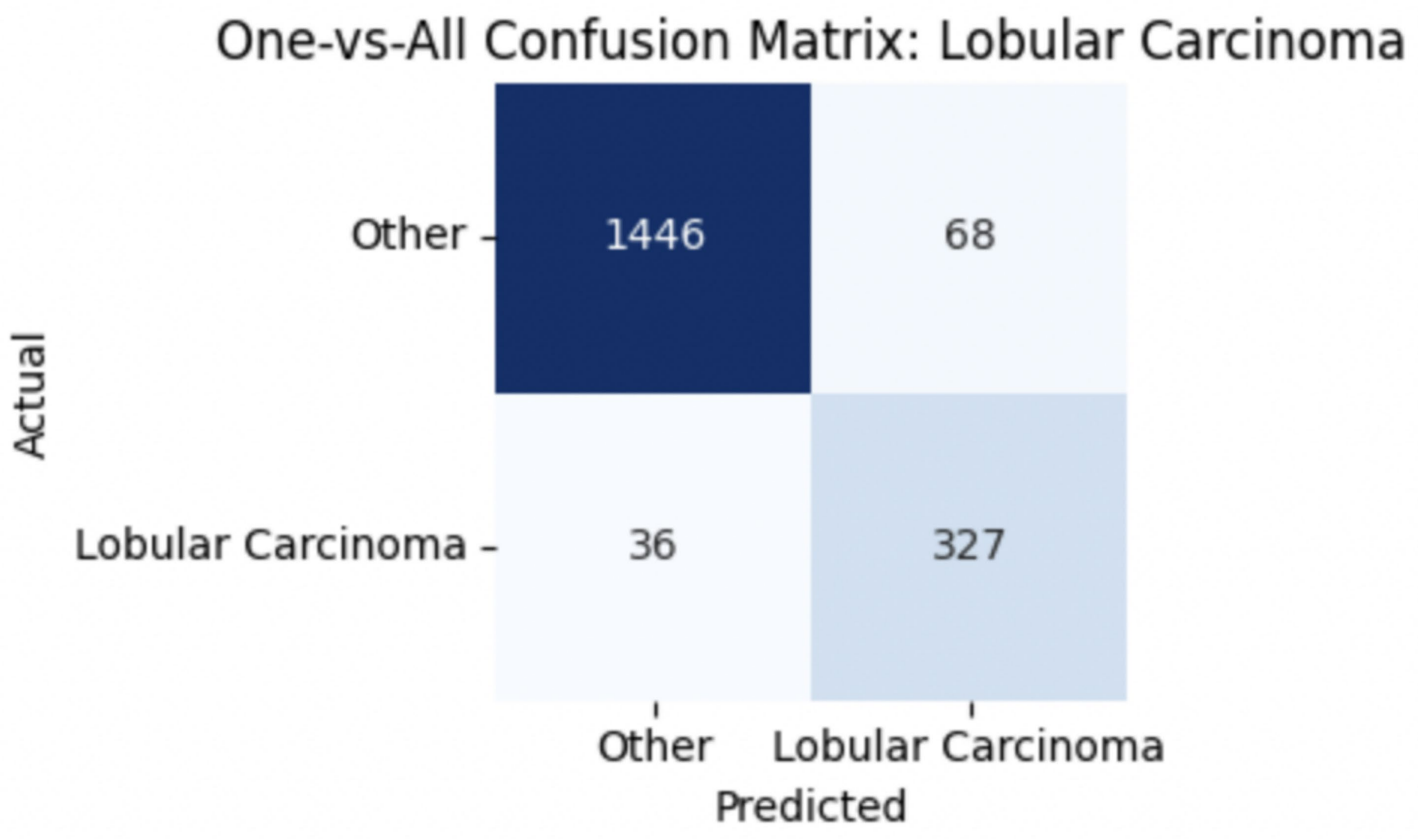
Lobular carcinoma confusion matrix.

**Figure 20:**
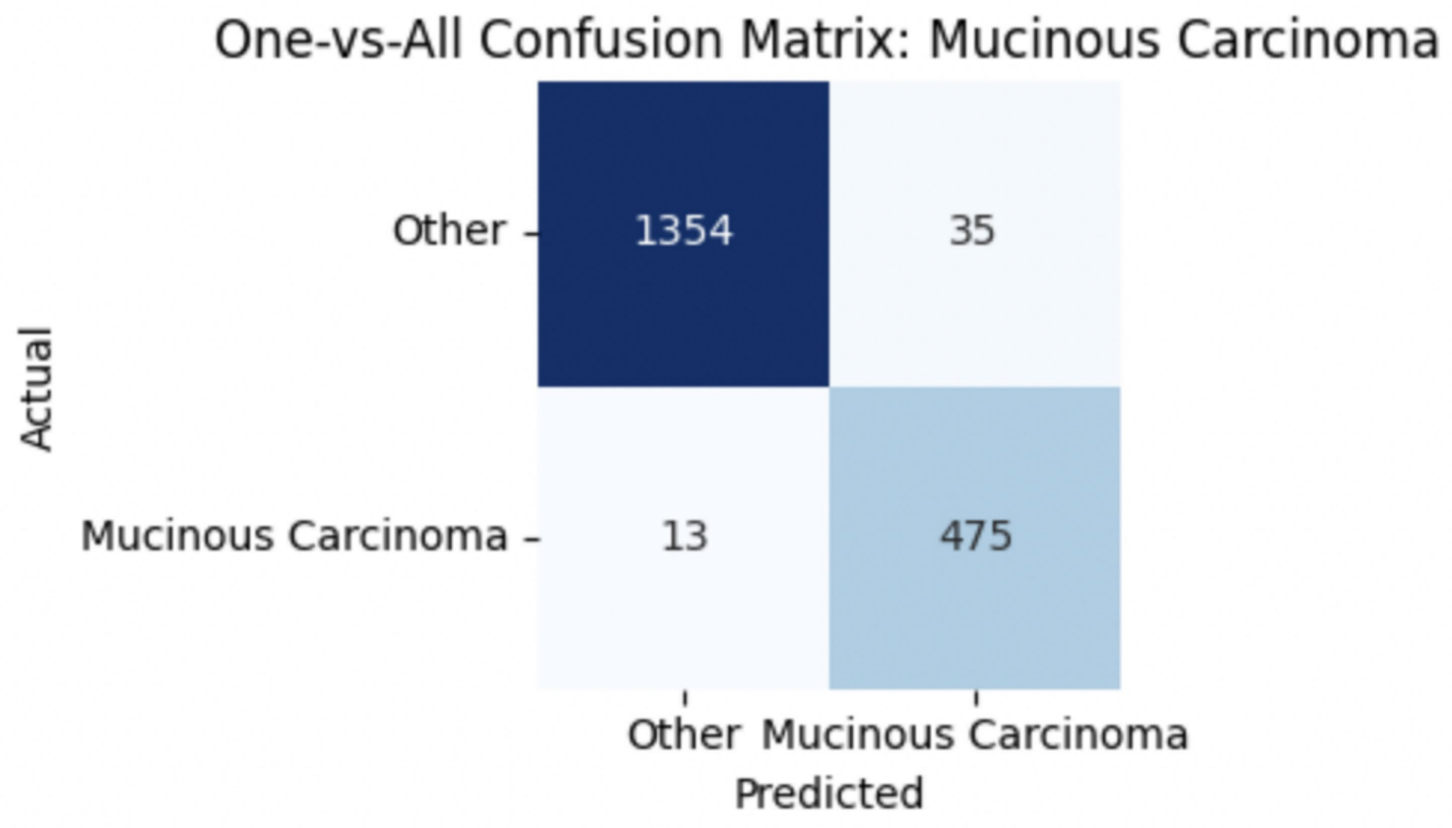
Mucinous carcinoma confusion matrix.

**Figure 21:**
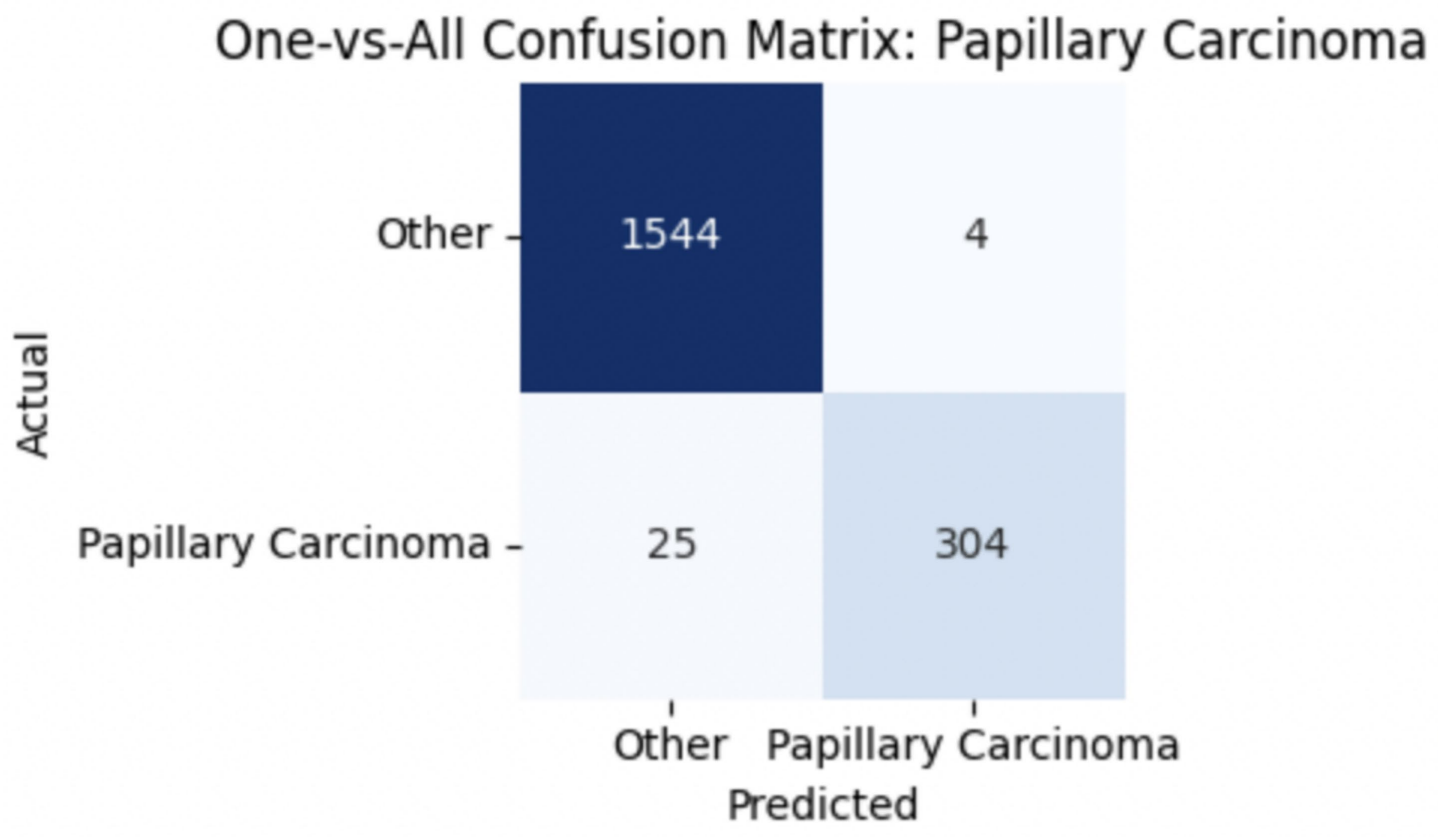
Papillary carcinoma confusion matrix.

For malignant tumor classification, per-class evaluation similarly revealed strong and balanced performance (see Table 13). The model classified mucinous carcinoma and PC with the highest F1-scores (both 0.95), supported by near-perfect precision and recall. DC, the most common subtype, achieved an F1-score of 0.91 with strong precision (0.93) and recall (0.89). The relatively lower performance was observed for LC, with an F1-score of 0.86, primarily due to reduced precision (0.83), indicating overlap in predicted labels with other malignant types. These distinctions are clearly reflected in the one-vs-all confusion matrices, which reveal class-specific prediction errors and highlight the model’s ability to distinguish even closely related malignancies. This subclass-level analysis enhances transparency and helps identify where architectural or dataset-level adjustments may be necessary for further improvement. Figures 18–21 show the confusion matrices for Ductal Carcinoma, Lobular Carcinoma, Mucinous Carcinoma, and Papillary Carcinoma, respectively.

**Table 13:** Per-class metrics for malignant classes.

Classification task	Precision	Recall	F1-score
Ductal carcinoma	0.93	0.89	0.91
Lobular carcinoma	0.83	0.90	0.86
Mucinous carcinoma	0.93	0.97	0.95
Papillary carcinoma	0.99	0.92	0.95

Although this study primarily focuses on classification performance, model interpretability is essential for clinical adoption. Techniques such as gradient-weighted class activation mapping (grad-CAM) or SHapley additive exPlanations (SHAP) can be used to generate heatmaps that visualize which regions of a biopsy image most influenced the model’s predictions. These visual explanations could help verify whether the model is focusing on diagnostically relevant features—Such as nuclear pleomorphism, stromal arrangement, or mitotic activity—Thereby increasing clinician trust and facilitating integration into diagnostic workflows.

### 3.3 K-fold cross-validation and performance stability

To evaluate the generalizability of the proposed classifiers, we performed 5-fold cross-validation on each of the three tasks: binary classification, benign subtype classification, and malignant subtype classification. The mean values, standard deviations, and 95% confidence intervals are presented in Table 14.

**Table 14:** Cross-validation scores for each model.

Task	Metric	Score (±SD)	95% CI
Binary class	Accuracy	0.9712 ± 0.0069	0.9616–0.9808
	Precision	0.9874 ± 0.0064	0.9785–0.9964
	Recall	0.9608 ± 0.0160	0.9386–0.9830
	F1-score	0.9738 ± 0.0062	0.9652–0.9825
Benign subtype	Accuracy	0.9375 ± 0.0208	0.9087–0.9663
	Precision	0.9468 ± 0.0132	0.9284–0.9651
	Recall	0.9300 ± 0.0274	0.8919–0.9680
	F1-score	0.9369 ± 0.0203	0.9087–0.9650
Malignant subtype	Accuracy	0.9202 ± 0.0080	0.9091–0.9314
	Precision	0.9048 ± 0.0122	0.8878–0.9217
	Recall	0.8808 ± 0.0265	0.8440–0.9176
	F1-score	0.8907 ± 0.0157	0.8689–0.9125

As shown in Table 14, the proposed classifiers maintained strong and stable performance across 5-fold cross-validation, with F1-scores of 0.9738 for binary classification, 0.9369 for benign subtype classification, and 0.8907 for malignant subtypes. The relatively tight confidence intervals and low standard deviations across all tasks suggest minimal sensitivity to training split variability, indicating good model generalization. Notably, the benign subtype classifier showed slightly higher performance consistency than the malignant subtype model, which exhibited greater variance in recall (±0.0265) and a broader 95% CI (0.8440–0.9176). This is likely due to the greater morphological diversity and class imbalance among malignant categories—particularly LC—where lower sample size and higher inter-class overlap may amplify prediction uncertainty.

The observed decline in recall and F1-scores compared to the single train-test split is expected, and it reflects the increased rigor of cross-validation, which exposes the model to harder-to-classify samples in more diverse folds. From a diagnostic perspective, the relatively stable precision across tasks implies that the model is conservative in positive predictions, minimizing false positives—an important trait in histopathological screening scenarios. However, the slightly larger variation in recall for rare or borderline subtypes warrants attention, as it may affect sensitivity to clinically ambiguous cases.

These findings reinforce the utility of k-fold cross-validation as a stress-testing tool, revealing not just average performance but also subtype-specific reliability under different sampling conditions. Future iterations of the model could incorporate stratified sampling by subtype or uncertainty-aware training strategies to further reduce variability in underrepresented classes.

### 3.4 Final notes

The final model, built on DenseNet121 with multi-scale feature extraction, demonstrated the strongest performance among all evaluated architectures, achieving high accuracy, recall, and F1-scores in the binary classification of breast cancer biopsy images. By extracting and fusing features from three intermediate convolutional blocks, the model was able to learn both fine-grained cellular structures and broader tissue-level patterns relevant to histopathological classification. The incorporation of L2 normalization, dropout regularization, and batch normalization within each branch further contributed to its stability and generalization. This design effectively addressed limitations observed in earlier models, such as feature loss due to single-layer reliance or overfitting from insufficient regularization.

Compared to existing models evaluated on the same BreaKHis dataset, the proposed approach offers a substantial improvement. Araújo et al. (2017) employed a standard CNN and achieved an average accuracy of 83.3% across all magnification levels. Similarly, Vo et al. (2019) reported 86.3% accuracy using a handcrafted feature pipeline with SVMs, while Bayramoglu et al. (2016) applied a multi-scale CNN and reached approximately 88.0% accuracy. More recently, Munshi et al. (2024) proposed an ensemble CNN-SVM framework with XAI components, achieving 94.2% accuracy and an F1-score of 0.93. In contrast, our DenseNet121-based model consistently achieved >97% accuracy and an F1-score of 0.9738 in binary classification, while maintaining robust generalization across folds and magnifications. These improvements can be attributed to the deeper architectural depth, feature reuse enabled by dense connectivity, and the incorporation of multi-scale fusion, which allowed the model to capture both cellular and architectural histological patterns more effectively.

The model’s strong and consistent performance across both binary and subtype classification tasks positions it as a scalable and technically rigorous approach for digital histopathology. With additional validation on multi-institutional and heterogeneous datasets, this framework has the potential to contribute meaningfully to diagnostic support systems for breast cancer, especially in settings with limited expert pathologist availability.

## 4 Conclusion

This study presents a deep learning framework for breast cancer diagnosis that performs subtype-level classification directly from H&E-stained biopsy images using a DenseNet121-based multi-scale feature fusion architecture. In conventional diagnostic workflows, the determination of histological subtypes often requires additional procedures—such as immunohistochemistry (IHC), molecular assays, or serial imaging—that increase diagnostic latency, invasiveness, and healthcare costs (Robbins et al., 2010; National Cancer Institute, 2021; Manning et al., 2018). In contrast, our proposed model offers a streamlined, image-only approach capable of distinguishing both benign and malignant lesions and further subclassifying them into clinically relevant subtypes (Spanhol et al., 2016; Rakhlin et al., 2018).

Our novel methodological contributions allow the model to capture both fine-grained cellular features and broader tissue-level patterns in a unified, end-to-end framework. Notably, this design improves over standard transfer learning approaches that rely solely on final-layer representations or require external fusion mechanisms (Gupta and Bhavsar, 2018; Zhu et al., 2019; Wakili et al., 2022).

While the model achieved strong binary classification accuracy (98.63%) and high F1-scores in multi-class subtype tasks (benign: 0.9415, malignant: 0.9251), its clinical utility is best positioned in supporting difficult diagnostic cases—such as distinguishing PTs from fibroadenomas or mucinous from PCs—where visual overlap challenges even experienced pathologists (Barker et al., 2018; Thompson et al., 2019). The model’s robustness across 5-fold cross-validation further reinforces its potential reliability in diverse settings (Kohavi, 1995; Berrar, 2019).

We emphasize that this tool is not a replacement for pathologists but a potential assistive technology to aid in triaging, second-opinion support, and prioritizing ambiguous cases. In resource-limited settings where access to expert pathology or advanced molecular testing is constrained, such a tool could help reduce diagnostic bottlenecks and improve equity in care delivery (McKinney et al., 2020; WHO, 2021).

However, further validation on larger and more heterogeneous datasets is necessary before clinical deployment (Liew et al., 2021). Moreover, the integration of model interpretability mechanisms—such as Grad-CAM visualizations—remains an essential next step to enhance transparency and foster clinical trust (Xie et al., 2020; Lee et al., 2024).

## 5 Limitations and future work

While the proposed model demonstrates strong performance in both binary and subtype-level breast cancer classification, several limitations must be acknowledged. First, the model was trained and validated solely on the BreaKHis dataset, which—despite its popularity in computational pathology research—is limited in scale and diversity. Its 7,909 images come from only 82 patients, restricting the model’s generalizability across varied populations, staining protocols, and imaging equipment. Future studies should aim to validate the model on larger, multi-institutional datasets that capture real-world clinical variability to increase the generalizability of the study.

Second, the model’s predictions are based exclusively on morphological features extracted from H&E-stained slides and do not incorporate molecular information, such as Human Epidermal Growth Factor Receptor 2 (HER2), Estrogen Receptor / Progesterone Receptor (ER/PR), or triple-negative status. These receptor-level biomarkers are critical for treatment planning, and their exclusion limits the clinical applicability of the system. Expanding the model to include IHC images or genomic profiles would enable a more comprehensive diagnostic tool aligned with current oncology workflows.

Another limitation lies in the model’s use of single-magnification images during training, despite the fact that pathologists typically examine biopsies at multiple magnification levels to evaluate both cellular and tissue-level structures. Although our multi-scale feature extraction within DenseNet121 captures some hierarchical information, it does not replicate the diagnostic reasoning derived from viewing across magnifications. Future work could explore multi-resolution input strategies or hierarchical CNNs to better reflect clinical interpretation.

Moreover, the current model lacks interpretability features, which are increasingly essential for clinical integration. Tools such as Grad-CAM or SHAP could be used to highlight regions of interest in biopsy images, helping pathologists understand model decisions and assess reliability. Including these visual explanations would significantly enhance trust and transparency in real-world applications.

Additionally, while this study emphasizes a novel architecture, it does not provide comparative results against widely used CNN baselines such as VGG16, ResNet50, or EfficientNet, nor does it present ablation experiments isolating the impact of architectural components like L2 normalization or feature fusion. Including these comparisons would strengthen claims of architectural innovation and clarify which design elements drive performance gains.

Finally, deployment considerations remain speculative. The model has not yet been tested in live clinical settings or integrated into diagnostic workflows, where computational constraints, system latency, and compatibility with laboratory information systems pose practical challenges. Future work should also consider automating the augmentation pipeline—currently hand-tuned—using approaches such as AutoAugment or Generative Adversarial Network (GAN)-based synthesis to improve performance in rare subtypes and low-data scenarios.

By addressing these limitations through external validation, multimodal expansion, improved interpretability, and clinical simulation, this work can move closer to real-world deployment as a reliable assistive tool in digital breast pathology.

## Data Availability

The original contributions presented in the study are included in the article/supplementary material; further inquiries can be directed to the corresponding author.
